# Conservation of symbiotic signaling since the most recent common ancestor of land plants

**DOI:** 10.1073/pnas.2408539121

**Published:** 2024-12-31

**Authors:** Tatiana Vernié, Mélanie Rich, Tifenn Pellen, Eve Teyssier, Vincent Garrigues, Lucie Chauderon, Lauréna Medioni, Fabian van Beveren, Cyril Libourel, Jean Keller, Camille Girou, Corinne Lefort, Aurélie Le Ru, Yves Martinez, Didier Reinhardt, Kyoichi Kodama, Shota Shimazaki, Patrice Morel, Junko Kyozuka, Malick Mbengue, Michiel Vandenbussche, Pierre-Marc Delaux

**Affiliations:** ^a^Laboratoire de Recherche en Sciences Végétales, Université de Toulouse, CNRS, Université Toulouse III Paul Sabatier, Institut National Polytechnique Toulouse, Castanet-Tolosan 31320, France; ^b^Fédération de Recherche 3450, Plateforme Imagerie, Pôle de Biotechnologie Végétale, Castanet-Tolosan 31320, France; ^c^Department of Biology, University of Fribourg, Fribourg 1700, Switzerland; ^d^Graduate School of Life Sciences, Tohoku University, Sendai, Miyagi 980-8577, Japan; ^e^Laboratoire Reproduction et Développement des Plantes, Univ Lyon, ENS de Lyon, Université Claude Bernard Lyon 1, CNRS, l’Institut National de Recherche pour l'Agriculture, l‘alimentation et l‘Environnement, Lyon 69342, France

**Keywords:** evolution, plant symbiosis, *Marchantia*, arbuscular mycorrhizal symbiosis

## Abstract

Plants have colonized lands 450 million years ago. This terrestrialization was facilitated by developmental and functional innovations. Recent evo-devo approaches have demonstrated that one of these innovations was the mutualistic arbuscular mycorrhizal symbiosis (AMS). The genetic pathways that have been involved in the establishment and functioning of AMS since its evolution remain poorly described. Here, we found that intracellular colonization by AM fungi induces a transcriptional reporter of the common symbiosis pathway, well-described in angiosperms, in the liverwort *Marchantia paleacea*. Mutants of either of the three main genes of this pathway, *SYMRK*, *CCaMK,* and *CYCLOPS,* disrupt the ability of *M. paleacea* to associate with AM fungi. Finally, overexpressing gain-of-function CCaMK or CYCLOPS leads to convergent transcriptomic signatures that partially overlap with AMS. Altogether, our data indicate that plants have maintained three genes of the common symbiotic pathway to support symbiotic interactions since their most recent common ancestor.

## Significance

Symbioses have evolved in all lineages of the tree of life. Among them, the arbuscular mycorrhizal symbiosis (AMS) evolved in the first plants that colonized land 450 million years ago. The mechanisms that have allowed this very ancient symbiosis to evolve and to be maintained remain poorly described. Here, we demonstrate that *Marchantia paleacea* shares a signaling pathway with flowering plants. This strongly suggests that all extant land plants share this pathway for activating a dedicated genetic program in the presence of the arbuscular mycorrhizal symbiont. Like in flowering plants, deleting this pathway in *M. paleacea* is sufficient to completely abolish symbiosis. We propose that plants have maintained a signaling pathway to support symbiosis for 450 million years.

The colonization of land by plants 450 million years ago revolutionized life on Earth (1). Identifying the key developmental and functional innovations that plants have evolved to deal with this new environment has been a long-standing question in evolutionary biology. It has been long postulated that terrestrialization was facilitated by the mutualistic interactions with symbiotic arbuscular mycorrhizal fungi, called Arbuscular Mycorrhizal Symbiosis [AMS (2, 3)]. Mechanisms allowing this symbiosis are well described in angiosperms and the development of a new plant model, *Marchantia paleacea,* recently allowed to investigate the conservation of some of them. It was shown for example that plant–fungus carbon fluxes rely on a conserved lipid delivery pathway (2) and that strigolactones, a molecule that has a dual function in plant–fungus communication and as plant hormone in angiosperms, functions solely as a trans-kingdom mode of communication in *M. paleacea* (4). Such comparisons of bryophytes and angiosperms allow both to assess the conservation of symbiotic mechanisms and to reconstruct the ancestral symbiosis that existed in the last common ancestor of land plants, following the evo-devo conceptual framework. This framework allows reconstructing trait and pathway evolution through different complementary steps relying on knowledge in extant species, including phylogenetics, trans-complementation assays, and reverse genetics (5). In particular, it allows i) determining the conservation of the biological function of genes and pathways, by comparing the phenotypes of the respective mutants in diverse species, and ii) testing the conservation of the molecular function in a particular context by conducting trans-complementation assays and expression in heterologous systems.

Hallmark of plant intracellular symbioses, the Common Signaling Pathway (CSP) had long been studied in angiosperms including dicots such as *Medicago truncatula* and monocots such as rice (6). The central components including the receptor-like kinase SYMRK/DMI2 (7, 8), the Calcium and Calmodulin-dependent protein kinase CCaMK/DMI3 (9), and the transcription factor CYCLOPS/IPD3 (10–12) had been shown to be indispensable for the establishment of both AM symbiosis and root nodule symbiosis in angiosperms, earning the name CSP (13). Phylogenomics conducted on the entire land plant lineage suggests that the ancestral function of those three genes was associated to AMS, and that they were recruited in more recently evolved intracellular symbioses (14, 15). However, the role of the CSP outside of angiosperms had never been investigated.

In this study, we demonstrate that activation of the CSP occurs in the bryophyte *M. paleacea* like in the angiosperms. We demonstrate that SYMRK/DMI2, CCaMK/DMI3, and CYCLOPS/IPD3 are essential for colonization by AM fungi in *M. paleacea*. Together our data indicate that the signaling pathway composed by those three genes has been dedicated to supporting symbiotic interactions since the last common ancestor of land plants.

## Results

### The CYCLOPS-Responsive Element Is a Marker of CSP Induction.

**Fig. 1.**
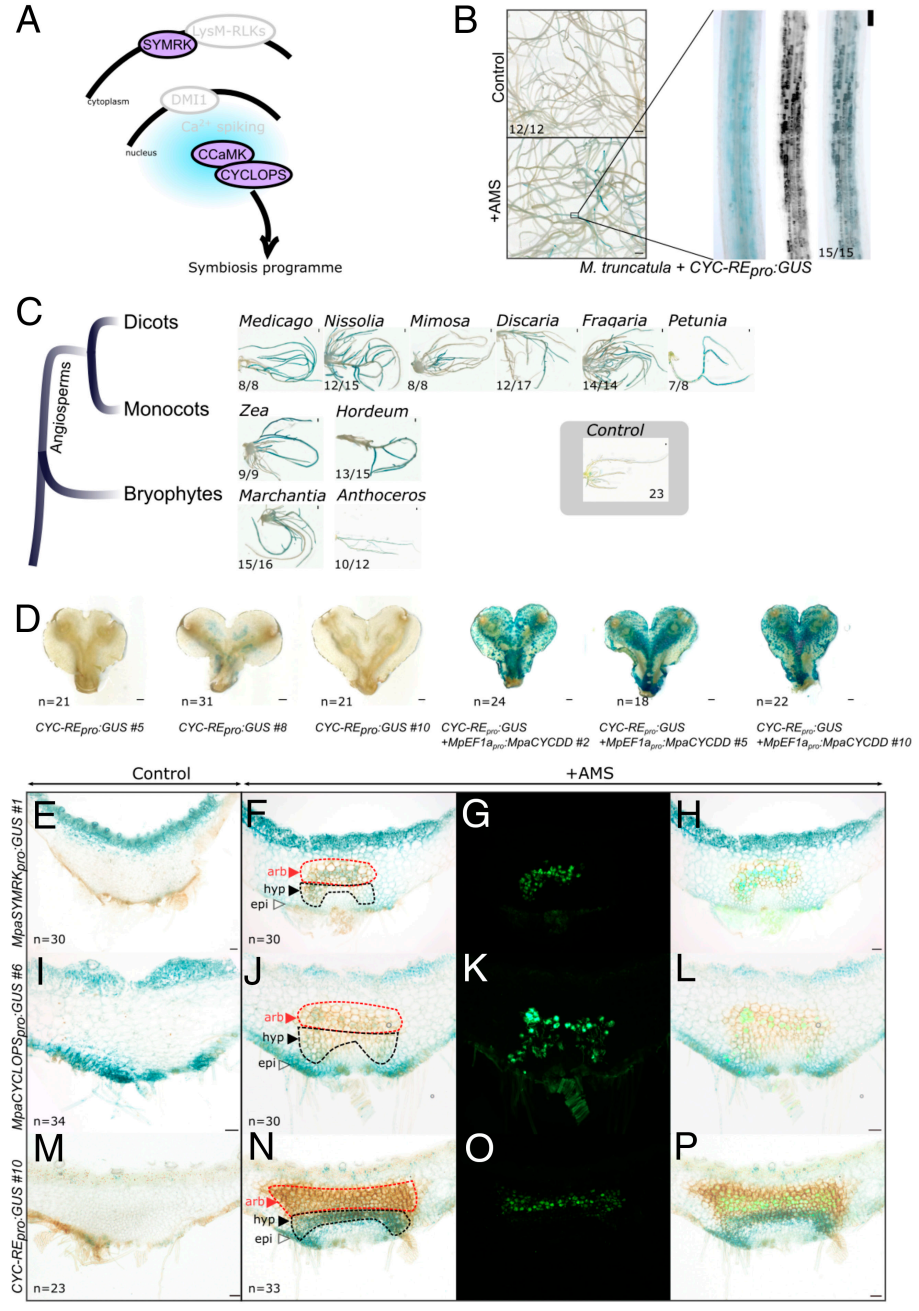
Conservation of *CYC-RE*_*pro*_*:GUS* activation by the CSP transcription factor CYCLOPS in land plants. (*A*) Common Symbiosis Pathway (CSP) in angiosperms. (*B*) *CYC-RE_pro_:GUS* is activated in response to AMS in *M. truncatula* six weeks post inoculation with *R. irregularis*. Whole roots images of GUS-stained inoculated and noninoculated roots are shown (Scale bar, 1 mm.) Faint blue signals could be observed on noninoculated roots, whereas intense blue patches were observed on inoculated roots. The zoomed image corresponds to one of these blue patches with a WGA-Alexa Fluor 488 staining revealing AM fungi (black) and an overlay of both images. (Scale bar, 100 µm.) The number of plants showing these responses are indicated out of the number of plants transformed. (*C*) *M. truncatula* roots were transformed with *CYC-RE_pro_:GUS* and autoactive forms of CYCLOPS (CYCLOPS-DD) orthologs from *Medicago truncatula*, *Nissolia schottii*, *Mimosa pudica*, *Discaria trinervis*, *Fragaria vesca*, *Petunia axillaris*, *Zea mays*, *Hordeum vulgare*, *Marchantia paleacea*, and *Anthoceros agrestis* and stained for GUS activity. Control plants correspond to plants transformed only with *CYC-RE_pro_:GUS*. Plants showing a strong GUS signal out of the total number of observed plants are indicated. For the control, only a faint GUS signal was sometimes observed in the 23 plants observed. (Scale bar, 1 mm.) (*D*) *M. paleacea* transformed lines expressing *CYC-RE_pro_:GUS* (three independent lines: *CYC-RE_pro_:GUS #5*, *8* and *10*) or *CYC-RE_pro_:GUS* + *MpEF1a*_pro_*:MpaCYCLOPS-DD* (three independent lines: *#2*, *5,* and *10*) are shown after staining for GUS activity. (Scale bar, 1 mm.) Total number of thalli observed in three independent assays are indicated. (*E*–*H*) *M. paleacea* transformed with *MpaSYMRK_pro_:GUS* without inoculation (Control) or six weeks post inoculation with *R. irregularis*. Thalli were GUS stained and *R. irregularis* is visualized with WGA-Alexa Fluor 488. Bright field (*E* and *F*), Alexa Fluor 488 (*G*), and overlay (*H*) are shown. (Scale bar, 100 µm.) One line out of two is shown. Both showed the same expression pattern. Total number of thalli observed in three independent assays are indicated. Arrowheads indicate the lower epidermis (epi), the hyphae-containing area (hyp, black-dotted line), and the arbuscule area (arb, red-dotted line). (*I*–*L*) *M. paleacea* transformed with *MpaCYCLOPS_pro_:GUS* without inoculation (Control) or six weeks post inoculation with *R. irregularis*. Thalli were GUS stained and *R. irregularis* is visualized with WGA-Alexa Fluor 488. Bright field (*I* and *J*), Alexa Fluor 488 (*K*), and overlay (*L*) are shown. (Scale bar 100 µm.) One line out of three is shown. They all showed the same expression pattern. Total number of thalli observed in three independent assays are indicated. Arrowheads indicate the lower epidermis (epi), the hyphae-containing area (hyp, black-dotted line), and the arbuscule area (arb, red-dotted line). (*M*–*P*) *M. paleacea* transformed with *CYC-RE_pro_:GUS* (*CYC-RE_pro_:GUS #10*) without inoculation (Control) or six weeks post inoculation with *R. irregularis*. Thalli were GUS stained and *R. irregularis* is visualized with WGA-Alexa Fluor 488. Bright field (*M* and *N*), Alexa Fluor 488 (*O*), and overlay (*P*) are shown. (Scale bar, 100 µm.) One line out of three is shown. They all showed the same expression pattern. Total number of thalli observed in three independent assays are indicated. Arrowheads indicate the lower epidermis (epi), the hyphae-containing area (hyp, black-dotted line), and the arbuscule area (arb, red-dotted line).

In angiosperms, activation of the CSP (Fig. 1*A*) upon symbiont perception leads to the phosphorylation of the transcription factor CYCLOPS by CCaMK, and the transcriptional activation of its direct target genes (12, 16). This direct activation by phosphorylated CYCLOPS is mediated by *cis-*regulatory elements present in the promoter region of the target genes (12, 16). The first of the four different *cis-*regulatory elements bound by phosphorylated CYCLOPS described so far (12, 16–18) was identified in the promoter of *NIN* from the angiosperm *Lotus japonicus*. A fusion of this element to a GUS reporter (*CYC-RE_pro_:GUS*) is activated during infection by rhizobial symbionts forming the root-nodule symbiosis in *L. japonicus* (12, 19) and *M. truncatula* (SI Appendix, Fig. S1*A*). We hypothesized that this reporter could be directly activated by phosphorylated CYCLOPS irrespective of the symbiotic context, and not specifically during the root-nodule symbiosis. *M. truncatula* hairy-roots transformed with the *CYC-RE_pro_:GUS* reporter were grown in presence or absence of the Arbuscular Mycorrhizal (AM) fungus *Rhizophagus irregularis*, harvested 6 wk after inoculation and stained for GUS activity. By contrast with the noninoculated roots that showed only faint and rare staining, the plants inoculated with *R. irregularis* showed consistent and intense GUS induction (Fig. 1*B* and SI Appendix, Fig. S1 *B*–G). The intense staining colocalized with the presence of the fungal hyphae and arbuscules (Fig. 1*B*). The *CYC-RE_pro_:GUS* reporter is thus a marker for the activation of the CSP by rhizobial and AM fungi symbionts in *M. truncatula.*

To determine whether this activation is specific to the phosphorylation of MtCYCLOPS, we coexpressed in *M. truncatula* hairy roots the *CYC-RE_pro_:GUS* reporter together with versions of CYCLOPS from various species mimicking the phosphorylation by CCaMK [CYCLOPS-DD (12)]. Overexpression of *CYCLOPS-DD* from *M. truncatula, Mimosa pudica,* or *Discaria trinervis* which are all able to form both the root-nodule and AM symbioses induced the *CYC-RE_pro_:GUS* reporter in *M. truncatula* hairy roots (Fig. 1*C*). The same activation was observed when overexpressing *CYCLOPS-DD* from *Fragaria vesca* and *Nissolia schottii* which belong to lineages that lost the ability to form the root-nodule symbiosis, but retained the AM symbiosis (20). Finally, CYCLOPS-DD from the AM-hosts dicot *Petunia axillaris*, monocots *Zea mays* and *Hordeum vulgare*, and bryophytes *M. paleacea* (thalloid liverworts) and *Anthoceros agrestis* (hornworts), all induced the *CYC-RE_pro_:GUS* reporter in the absence of AM fungi (Fig. 1*C*). Activation of the *CYC-RE_pro_:GUS* is thus not limited to phosphomimetic CYCLOPS from species able to form both the root-nodule and the AM symbioses.

To determine whether the activation of the *CYC-RE_pro_:GUS* was dependent on the genetic background or the symbiotic abilities of the plant species, we expressed *MtCYCLOPS-DD* and the *CYC-RE_pro_:GUS* reporter in the root of the legume *Nissolia brasiliensis*. The genus *Nissolia* has lost the ability to form the root-nodule symbiosis but retains the AM symbiosis (20). As for the expression in *M. truncatula,* overexpression of *MtCYCLOPS-DD* resulted in the activation of the *CYC-RE_pro_:GUS* reporter in *N. brasiliensis* (SI Appendix, Fig. S1 *H*–I). Finally, the same experiment was conducted in the liverworts *M. paleacea*. Among bryophytes, the liverwort *M. paleacea* is able to engage in AMS (15, 21) and has emerged as an appropriate model to study the conservation of symbiotic processes in land plants. Overexpression of *MpaCYCLOPS-DD* leads, similarly, to the activation of the *CYC-RE_pro_:GUS* reporter (Fig. 1*D*), while control lines only expressing the *CYC-RE_pro_:GUS* reporter did not show staining, or only faint staining (Fig. 1*D*).

Collectively, these data indicate that the *CYC-RE_pro_:GUS* is a reliable marker for the activation of the CSP, irrespective of the plant species and the type of symbiosis.

### The CSP Is Activated During Symbiosis in *M. paleacea*.

The bryophyte and vascular-plant lineages diverged *ca.* 450 million years ago (22). Because of this early split during land-plant evolution, identifying conserved features between representatives of these two lineages allows inferring the biology of their most recent common ancestor, a close relative of the first land plants (22). To determine whether the activation of the CSP is conserved across land plants, we first transformed *M. paleacea* with *promoter:GUS* fusions for the upstream- and downstream-most components of the CSP, namely *SYMRK* and *CYCLOPS*. The lines were inoculated with *R. irregularis* or mock-treated, harvested six weeks later, and stained. In noninoculated conditions, the *MpaSYMRK_pro_:GUS* and *MpaCYCLOPS_pro_:GUS* lines displayed background staining in the upper and lower epidermis. In *M. paleacea*, AM fungi colonize through the rhizoids, reach the lower part of the thallus where they develop intracellular hyphea and colonize the parenchyma. Arbuscules are formed in a specific area of the parenchyma that accumulates a purple pigment upon infection (Fig. 1). Upon inoculation, both *MpaSYMRK_pro_:GUS* and *MpaCYCLOPS_pro_:GUS* displayed an additional expression domain in the cells hosting arbuscules and in the area just below, where intracellular hyphae develop (Fig. 1 *F* and *J*).

To directly test the link between AM symbiosis and the CSP, the *M. paleacea CYC-RE_pro_:GUS* reporter lines were inoculated with *R. irregularis*, grown for six weeks and the activation of the reporter tested by GUS staining. While noninoculated plants showed barely any GUS signal, inoculated plants displayed a robust signal in the part of the thallus hosting intracellular hyphae (Fig. 1*N*). By contrast with *M. truncatula* roots, no signal was observed in the area hosting mature arbuscules, just above the intracellular hypheae (Fig. 1*N*).

These results indicate that the CSP is activated during intracellular colonization by AM fungi in *M. paleacea*.

### The Symbiotic Function of SYMRK Is Conserved Since the Most Recent Common Ancestor of Land Plants.

**Fig. 2.**
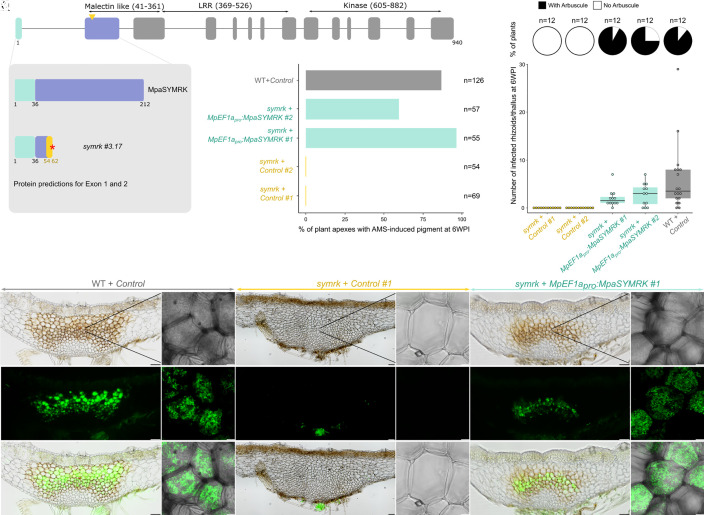
SYMRK is essential fo Arbuscular Mycorrhizal Symbiosis (AMS) in *M. paleacea.* (*A*) Exons/introns structure of the *MpaSYMRK* genomic sequence. The number of amino acids and predicted domains are indicated. CRISPR/Cas9 was conducted on exon 1 (arrowhead indicates position of sgRNAs). (*B*) Protein sequence predictions for the 2 first exons of *MpaSYMRK* in Wild type and *symrk #3.17* line. Red asterisks indicate the presence of a premature stop codon in the mutant line. (*C*) Colonization rates of *M. paleacea symrk* mutant lines at six weeks post inoculation with *R. irregularis*. Colonization rates are estimated by quantifying the presence of AMS-induced pigment in *M. paleacea* apexes of a wild type line transformed with an empty vector and *symrk #3.17* line transformed with an empty vector (control #1 & 2) or *MpEF1a_pro_:MpaSYMRK* (#1 & 2). Numbers of observed plant apexes quantified in two independent assays are indicated (n). (*D*) Number of infected rhizoids and presence of arbuscules scored at six weeks post inoculation with *R. irregularis* in wild type line transformed with an empty vector and *symrk #3.17* line transformed with an empty vector (control #1 & 2) or *MpEF1a_pro_:MpaSYMRK* (#1 & 2). Pie charts indicate the percentage of thalli with arbuscules and box plots the number of infected rhizoids in these thalli. Twelve thalli were observed for each line on two independent assays. (*E*–*V*) Transversal sections of wild-type line transformed with an empty vector (*E*, *F*, *K*, *L*, *Q*, and *R*) and *symrk #3*.17 line transformed with an empty vector (#1, *G*, *H*, *M*, *N*, *S*, and *T*) or *MpEF1a_pro_:MpaSYMRK* (#1, *I*, *J*, *O*, *P*, *U*, and *V*) six weeks post inoculation with *R. irregularis*. *R. irregularis* is visualized with WGA-Alexa Fluor 488. Close-up view of cells in the colonized area by confocal microscopy (*F, H, J, L, N, P, R, T*, and *V*). Bright field (*E*–*J*), Alexa Fluor 488 (*K*–*P*), and overlay (*Q*–*V*) are shown for each line. [Scale bar, 100 µm (Nikon microscopy) and 10 µm (confocal microscopy).]

The activation of a CYCLOPS-inducible reporter during AM symbiosis in *M. palacea* is yet another correlative evidence for the conserved symbiotic role of the CSP in land plants. To directly test this role, we generated nine *symrk* mutant alleles in *M. paleacea* using CRISPR/Cas9 (SI Appendix, Fig. S2). Four alleles lead to nonsense mutations coding for predicted truncated proteins (SI Appendix, Fig. S2). The other five alleles displayed missense mutations and small deletions that left the downstream original reading frame intact (SI Appendix, Fig. S2). The nine mutants were inoculated with *R. irregularis* in parallel with a line transformed with an empty vector (control line). While 96% of the control line thalli were colonized and showed arbuscules five weeks after inoculation, none of the four nonsense alleles lines showed signs of colonization (SI Appendix, Fig. S3). Among the five missense mutants two were not colonized (SI Appendix, Fig. S3) and the other three showed infection by *R. irregularis* but a strong quantitative reduction in colonization when assessed using the pigment produced by *M. paleacea* upon colonization by AMS as a proxy (SI Appendix, Fig. S3). Microscopy confirmed the colonization defect in the *symrk* mutant lines, and the presence of fully developed infection units harboring arbuscules in the control line (SI Appendix, Fig. S3). To further determine the function of SYMRK during AMS in *M. paleacea*, we retransformed one of the loss-of-function alleles with a *MpEF1a_pro_:MpaSYMRK* construct or an empty vector. The *MpEF1a_pro_* was used because it cannot be excluded that an essential regulatory element is missing from the 2 kb *MpaSYMRK* promoter. While the lines retransformed with the empty vector did not show any sign of colonization, either using the pigment as a proxy, or ink/WGA staining, two independent complemented lines had AMS restored (Fig. 2). To better characterize when AMS was affected in the *symrk* mutant, we scored the number of rhizoids infected by *R. irregularis*. While the control line and the complemented lines all harbored infected rhizoids, not a single infected rhizoid was observed in the *symrk* mutant (Fig. 2). The consistent AM symbiosis defect in the *M. paleacea symrk* mutant lines is similar to the phenotypes observed in diverse *symrk* mutant of dicots (7, 8) and monocots (23, 24) in which colonization by AM fungi ranges from a low level of colonization to being completely abolished.

Altogether, these data indicate that the biological role of SYMRK for the establishment of the AM symbiosis is conserved across land plants.

### The Symbiotic Function of the CCaMK/CYCLOPS Module Is Conserved Since the Most Recent Common Ancestor of Land Plants.

Downstream of SYMRK, CCaMK and CYCLOPS act as a module triggering the initial steps of the symbiotic response. In legumes and the monocots rice and barley, CCaMK is essential for AM symbiosis, while *cyclops* mutants display phenotypes ranging from strong reduction in colonization rate to the absence of AM fungi (10, 23, 25). Here, we added to the range of tested angiosperms *ccamk* and *cyclops* mutants from a dicot that do not form the root-nodule symbiosis, the Solanaceous species *P. hybrida*. Intracellular colonization was neither observed in the *ccamk* mutant nor in the *cyclops* mutant, while the wild-type siblings were well colonized (SI Appendix, Table S1) confirming the important role of CCaMK and CYCLOPS for AM symbiosis in angiosperms. Next, we generated thirteen *ccamk* and seven *cyclops* mutants in *M. paleacea* by CRISPR/Cas9 (SI Appendix, Figs. S4 and S5).

**Fig. 3.**
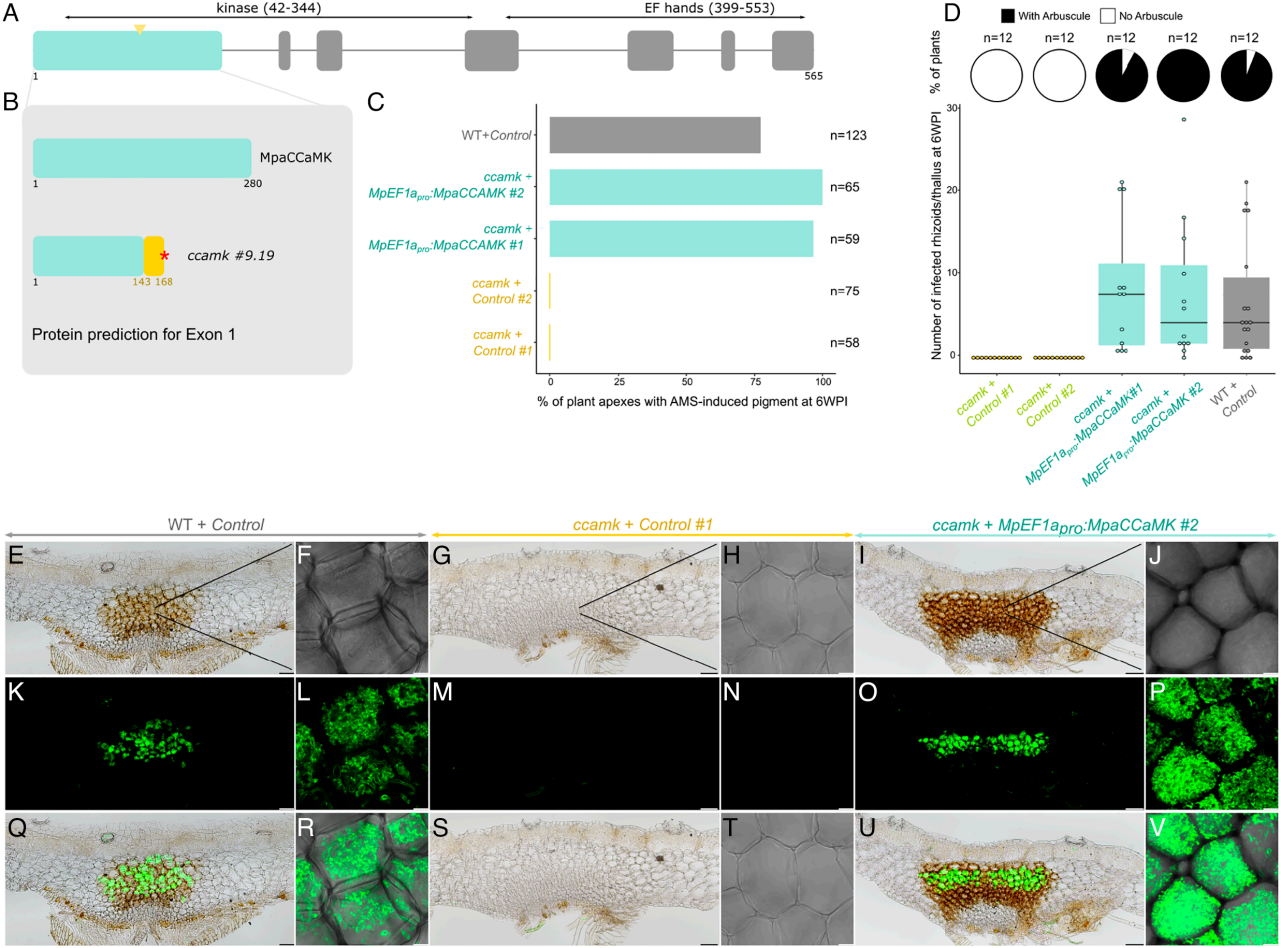
CCaMK is essential for AMS in *M. paleacea.* (*A*) Exons/introns structure of the *MpaCCaMK* genomic sequence. The number of amino acids and predicted domains are indicated. CRISPR/Cas9 was conducted on exon 1 (arrowhead indicates position of sgRNAs). (*B*) Protein sequence predictions for the first exon of *MpaCCaMK* in Wild type and *ccamk #9.19* line. Red asterisks indicate the presence of a premature stop codon in the mutant line. (*C*) Colonization rates of *M. paleacea ccamk* mutant lines at six weeks post inoculation with *R. irregularis*. Colonization rates are estimated by quantifying the presence of AMS-induced pigment in *M. paleacea* apexes of a wild-type line transformed with an empty vector and *ccamk #9.19* line transformed with an empty vector (control #1 & 2) or *MpEF1a_pro_:MpaCCaMK* (#1 & 2). Numbers of observed plant apexes quantified in two independent assays are indicated (n). (*D*) Number of infected rhizoids and presence of arbuscules scored at six weeks post inoculation with *R. irregularis* in wild type line transformed with an empty vector and *ccamk #9.19* line transformed with an empty vector (control #1 & 2) or *MpEF1a_pro_:MpaCCaMK* (#1 & 2). Pie charts indicate the percentage of thalli with arbuscules and box plots the number of infected rhizoids in these thalli. Twelve thalli were observed for each line on two independent assays. (*E*–*V*) Transversal sections of wild-type line transformed with an empty vector (*E, F, K, L, Q*, and *R*) and *ccamk #9.19* line transformed with an empty vector (#1, *G, H, M, N, S*, and *T*) or *MpEF1a_pro_:MpaCCaMK* (#2, *I, J, O, P, U*, and *V*) six weeks post inoculation with *R. irregularis*. *R. irregularis* is visualized with WGA-Alexa Fluor 488. Close-up view of cells in the colonized area by confocal microscopy (*F, H, J, L, N, P, R, T*, and *V*). Bright field (*E*–*J*), Alexa Fluor 488 (*K*–*P*), and overlay (*Q*–*V*) are shown for each line. [Scale bar, 100 µm (Nikon microscopy) and 10 µm (confocal microscopy).]

Following inoculation with *R. irregularis*, twelve of the *ccamk* mutants, showed no signs of colonization after five weeks, while the control line (44/54 thalli) and a *ccamk* mutant with only a small *in-frame* deletion in the sequence preceding the kinase domain (32/40 thalli) were normally colonized (SI Appendix, Fig. S6). Microscopy confirmed the absence of colonization in the *ccamk* mutant lines, and the presence of fully-developed infection units harboring arbuscules in the control line (SI Appendix, Fig. S6). Retransformation of one of the loss-of-function mutants with *MpEF1a_pro_:Mpa CCaMK* restored colonization in two independent lines, while retransformation with an empty vector did not (Fig. 3). While the control line and the complemented *ccamk* lines showed infected rhizoids, all of the observed *ccamk* mutant thallus had only noninfected rhizoids (Fig. 3). This demonstrates the essential symbiotic role of *CCaMK* in *M. paleacea*.

**Fig. 4.**
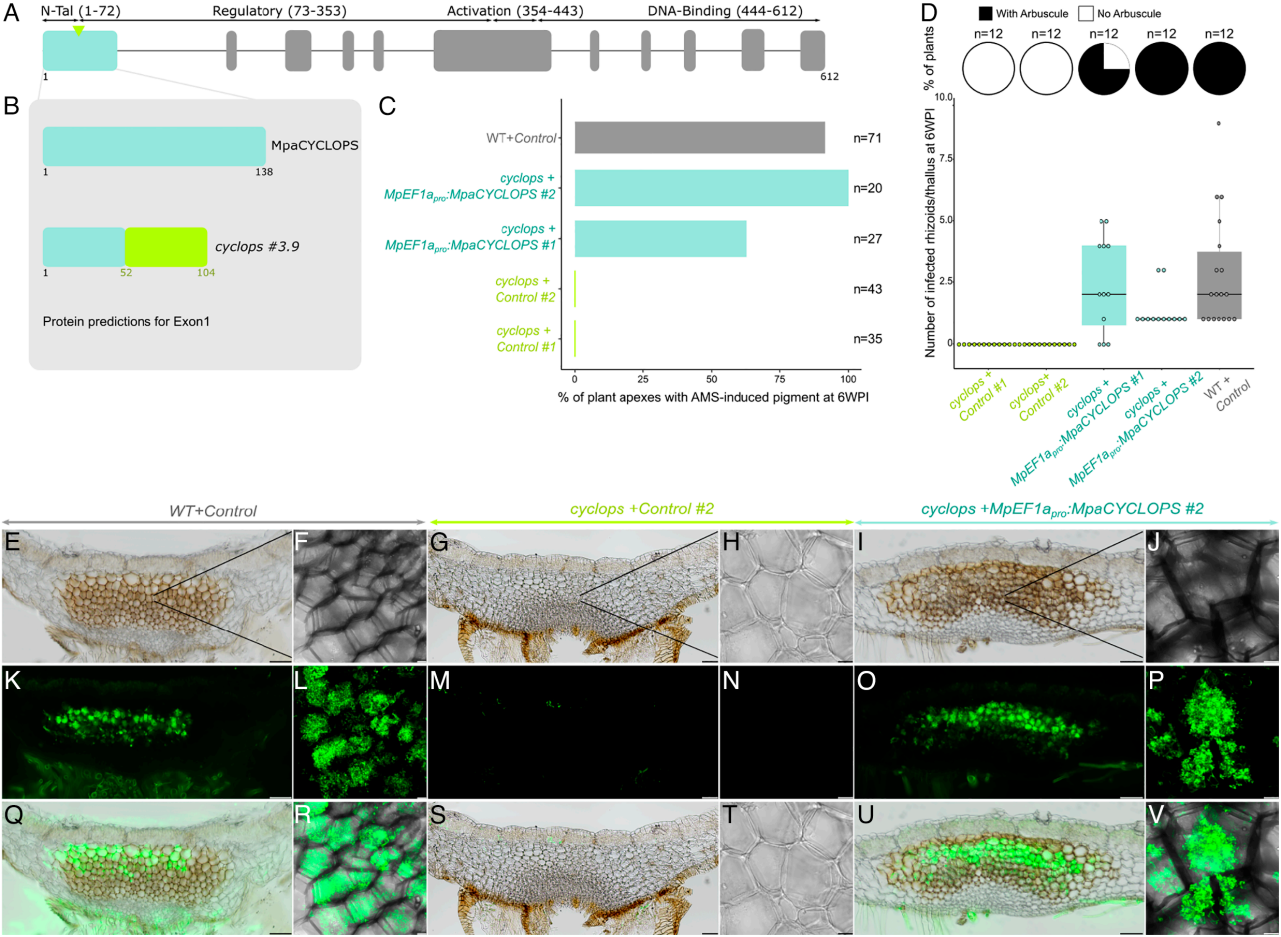
CYCLOPS is important for AMS in *M. paleacea.* (*A*) Exons/intron structure of the *MpaCYCLOPS* genomic sequence. Number of amino acids and predicted domains are indicated. CRISPR/Cas9 was conducted on exon 1 (arrowhead indicates position of sgRNAs). (*B*) Protein sequence predictions for the first exon of *MpaCYCLOPS* in Wild type and *cyclops #3.9* line. (*C*) Colonization rates of *M. paleacea cyclops* mutant lines at six weeks post inoculation with *R. irregularis*. Colonization rates are estimated by quantifying the presence of AMS-induced pigment in *M. paleacea* apexes of a wild-type line transformed with an empty vector and *cyclops #3.9* line transformed with an empty vector (control #1 & 2) or *MpEF1a_pro_:MpaCYCLOPS* (#1 & 2). Numbers of observed plant apexes quantified in two independent assays are indicated (n). (*D*) Number of infected rhizoids and presence of arbuscules scored at six weeks post inoculation with *R. irregularis* in wild-type line transformed with an empty vector and *cyclops #3.9* line transformed with an empty vector (control #1 & 2) or *MpEF1a_pro_:MpaCYCLOPS* (#1 & 2). Pie charts indicate the percentage of thalli with arbuscules and box plots the number of infected rhizoids in these thalli. Twelve thalli were observed for each line on two independent assays. (*E*–*V*) Transversal sections of wild-type line transformed with an empty vector (*E, F, K, L, Q*, and *R*) and *cyclops #3.9* line transformed with an empty vector (#2, *G, H, M, N, S*, and *T*) or *MpEF1a_pro_:MpaCYCLOPS* (#2, *I, J, O, P, U*, and *V*) six weeks post inoculation with *R. irregularis*. *R. irregularis* is visualized with WGA-Alexa Fluor 488. Close-up view of cells in the colonized area by confocal microscopy (*F, H, J, L, N, P, R, T*, and *V*). Bright field (*E*–*J*), Alexa Fluor 488 (*K*–*P*), and overlay (*Q*–*V*) are shown for each line. [Scale bar, 100 µm (Nikon microscopy) and 10 µm (confocal microscopy).]

Five weeks after inoculation with *R. irregularis*, the phenotypes of the *cyclops* frameshift mutants ranged from moderately to strongly reduced colonization, or to a total lack of colonization (SI Appendix, Fig. S7). One allele, only affected by a 12nt in-frame deletion, was colonized to a similar level than the control line when using the occurrence of the AMS-induced pigment as a proxy for colonization (SI Appendix, Fig. S7). Similar phenotypes were still observed eight weeks after inoculation (SI Appendix, Fig. S7). The observed difference in the strength of the phenotypes between *cyclops* mutants correlates with the position of the CRISPR/Cas9-induced mutations (SI Appendix, Fig. S7). While the alleles showing lack of, or strongly reduced, colonization were mutated in a domain conserved across all embryophytes (SI Appendix, Figs. S7 and S8) alleles with reduced colonization were mutated closer to the N-terminal part of the protein, in a domain conserved across bryophytes, ferns, and gymnosperms, but missing from the angiosperms (SI Appendix, Fig. S8). This additional domain may thus have a nonessential function. Presence of alternative start codons downstream the mutation present in the weak alleles (SI Appendix, Figs. S5 and S7) supports this hypothesis, although further experiments are required to test it. Strongly reduced colonization was also observed in two additional frameshift mutant lines generated in *M. paleacea ssp. diptera* (SI Appendix, Fig. S9). To consolidate the quantification of the phenotypes, RNA was extracted from the seven *M. paleacea cyclops* mutants and the empty vector control line five weeks after inoculation with *R. irregularis*, and the expression of the AM-responsive (2) phosphate (*MpaSymPT*) and lipid (*MpaSTR*) transporters was monitored by qRT-PCR. Expression of the *R. irregularis* housekeeping gene *RiTEF* was quantified as a proxy for fungal abundance. The expression level of *SymPT*, *STR,* and *RiTEF* mirrored the observed colonization rates defined based on the AMS-induced pigment (SI Appendix, Fig. S7). To further test the role of CYCLOPS, we retransformed the strong loss-of-function mutant with *MpEF1a_pro_:MpaCYCLOPS* or an empty vector. Two independent lines transformed with *MpEF1a_pro_:MpaCYCLOPS* showed recovery of AMS while the line transformed with the empty vector was not colonized (Fig. 4). Similar to the *symrk* and *ccamk* loss-of-function lines, rhizoids were not infected in the *cyclops* mutant, a phenotype complemented by the *MpEF1a_pro_:MpaCYCLOPS* construct (Fig. 4).

The early symbiotic defects observed in *M. paleacea ccamk* and *cyclops* mutants, reminiscent of the ones observed in angiosperms, indicate that the biological function of these two genes as regulators of the AM symbiosis is conserved across land plants.

### The Molecular Function of the CYCLOPS-CCaMK Module Is Conserved Since the Most Recent Common Ancestor of Land Plants.

**Fig. 5.**
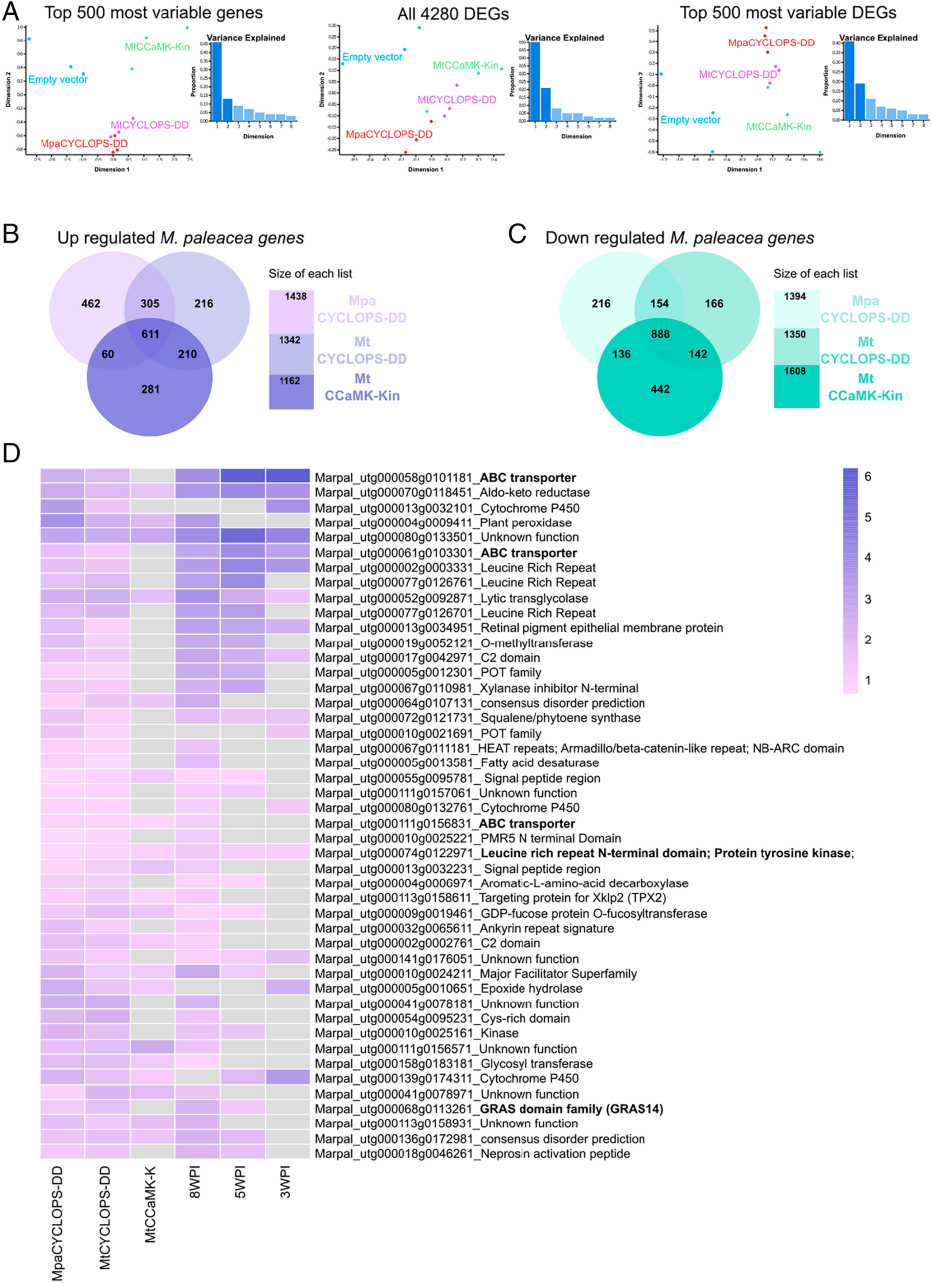
The CYCLOPS-CCaMK module is conserved in land plants. (*A*) Multidimensional scaling (MDS) analysis of the RNA-seq samples. Analysis run were performed on the 500 most variable genes across all samples, on the 4280 DEGs identified in Dataset S1 and on the 500 most variable from the DEGs identified in Dataset S1. Colors represent the different samples. (*B*) Venn diagrams of up-regulated genes in *M. paleacea* overexpressing *MpaCYCLOPS-DD*, *MtCYCLOPS-DD*, or *MtCCaMK-Kin* compared to control plants (FDR ≤ 0.05). (*C*) Venn diagrams of down regulated genes in *M. paleacea* overexpressing *MpaCYCLOPS-DD*, *MtCYCLOPS-DD*, or *MtCCaMK-Kin* compared to control plants (FDR ≤ 0.05). (*D*) Heatmap of log2 fold change of common *M. paleacea* genes in a time-course of AMF-inoculated compared with mock-treated plants, and in the *MpEF1a_pro_:MpaCYCLOPS-DD* and *MpEF1a_pro_:MtCYCLOPS-DD* overexpressing lines. (FDR ≤ 0.05). Boxes in gray correspond to an FDR>0.05.

Complementation of the symbiotic defects of angiosperms *ccamk* and *cyclops* mutants by their bryophyte orthologs supports the conservation of the molecular function of this module for AMS across plant lineages (14, 15, 26). In angiosperms, CCaMK phosphorylates CYCLOPS following symbiont-induced nuclear calcium spiking (12). In both *M. paleacea* and angiosperms expression of a gain of function version of *M. paleacea* CYCLOPS mimicking this phosphorylation (*MpEF1a_pro_*:*MpaCYCLOPS-DD*) leads to the activation of the *CYC-RE_pro_:GUS* reporter (Fig. 1 *C* and *D*). If the CYCLOPS/CCaMK module is indeed conserved across plant lineages, we reasoned that overexpression of *MpaCYCLOPS-DD*, *MtCYCLOPS-DD,* and the kinase domain of MtCCaMK—*MtCCaMK-Kin*, corresponding to MtCCaMK^1-311^, an autoactive version of CCaMK (27)—should result in similar transcriptomic signatures relative to control lines. To test this, *M. paleacea* lines overexpressing either of these three constructs were generated, their transcriptome determined by RNA-seq, and compared to lines transformed with an empty vector to identify differentially expressed genes (Fig. 5 and Dataset S1). All three constructs lead to very significant transcriptomic changes, ranging from 1,162 to 1,438 up-regulated genes, and 1,350 to 1,608 down-regulated genes (Fig. 5 and Dataset S1). The first component of a Principal Component Analysis separated the transcriptome of the empty vector lines from those of the *MpEF1a_pro_:CYCLOPS-DD* and *MpEF1a_pro_:CCaMK-Kin* lines (Fig. 5*A*). In this analysis, the expression of the most variable genes gathered the *MpEF1a_pro_:MtCYCLOPS-DD* and *MpEF1a_pro_:MpaCYCLOPS-DD* lines together, indicating that their transcriptomes are most similar. The similarity among the three CSP Gain-of-Function lines was further observed in the Principal Component Analysis when focusing on the top 500 DEGs (Fig. 5*A*). Among the genes up-regulated in response to the overexpression of *MpaCYCLOPS-DD,* 916 and 671 were also found up-regulated in response to *MtCYCLOPS-DD* and *MtCCaMK-Kin* respectively (Fig. 5*B*). A similar trend was observed for the down-regulated genes (1,042 and 1,024 respectively, Fig. 5*C*). These overlaps were significantly higher than expected by chance (SI Appendix, Table S2*C*) and corroborate the Principal Component Analysis. This result strongly supports the hypothesis that the molecular function of CYCLOPS and CCaMK in the context of AMS is conserved across land plants, although specificities probably evolved in each clade as illustrated by the not perfectly overlapping transcriptomic patterns.

To define the contribution of the CSP to AMS in *M. paleacea*, we cross-referenced the CYCLOPS-DD and/or CCaMK-Kin-regulated genes with previously obtained RNA-seq data in response to AM fungi^2^ (Dataset S1). Due to a lower sequencing depth, some of the genes lowly expressed in the AMS condition did not reach the threshold for DEG estimation in the overexpression experiment (Dataset S1). Nevertheless, 46 genes upregulated in the *MpEF1a_pro_:MtCYCLOPS-DD* and *MpEF1a_pro_:MpaCYCLOPS-DD* lines were found upregulated during AMS too (Fig. 5*D*), including 20 that were also up-regulated in the *MpEF1a_pro_:MtCCaMK-Kin* line. Among the genes shared between the *MpEF1a_pro_:Mpa CYCLOPS-DD* and *MpEF1a_pro_:MtCYCLOPS-DD* lines were several transporters, LRR-RLK, and one transcription factor of the GRAS family (GRAS14, Marpal_utg000068g0113261). Interestingly, among the few validated CYCLOPS targets in angiosperms is RAM1 (16), a GRAS transcription factor regulating arbuscule formation which has been lost in *M. paleacea* (15). Two additional GRAS transcription factors induced during AMS were specifically up-regulated in the *MpEF1a_pro_:MpaCYCLOPS-DD* lines (*GRAS 10* and *GRAS5*, SI Appendix, Table S2*B*). Furthermore, another GRAS transcription factor well known for its function as regulator of arbuscule formation in angiosperms, *RAD1* (28), and activated during AMS in the arbuscule-contained area in *M. paleacea* (2) was up-regulated in the *MpEF1a_pro_:MpaCCaMK-Kin* lines (Dataset S1). Altogether, this indicates that a network of GRAS transcription factors may act downstream of the CSP in *M. paleacea* like in angiosperms.

Mirroring the trans-complementation of angiosperm mutants with liverwort sequences (14, 15, 26), these results indicate that, similar to the conservation of the biological role of the CYCLOPS-CCaMK module, the molecular function of these two components of the CSP is conserved across land plants although the gene network they regulate likely diversified.

## Discussion

This work demonstrates that the three main CSP components SYMRK/DMI2, CCaMK/DMI3, and CYCLOPS/IPD3 are essential for AM symbiosis in the liverwort *M. paleacea* (Figs. 2–4), as described over the last two decades for angiosperms. To explain this conserved role, the most parsimonious hypothesis suggests that the most recent common ancestor of the angiosperms and the bryophytes already used the CSP to engage and associate with AM fungi. In other words, together with the recent description of the role played by the fourth CSP gene, *DMI1*, in AMS in *M. paleacea* (29) our data indicate that land plants have relied on a conserved symbiotic signaling pathway since their most recent common ancestor. From a symbiotic perspective to an evo-devo one, this finding opens up a number of questions.

Overexpression of CYCLOPS and CCaMK gain-of-function versions (CYCLOPS-DD and CCaMK-Kin) led to significant, and overlapping, gene induction in *M. paleacea* (Fig. 5). When comparing the list of DEGs in response to the overexpression of CYCLOPS-DD or CCaMK-Kin with the DEGs during AMS in *M. paleacea* the overlap differs. Such a difference might come from the more upstream position of CCaMK in the CSP which goes through CYCLOPS but also alternative side pathways to be described. Illustrating these bifurcations in the CSP, two Cyclin-dependent Kinase-Like (CKL1 and CKL2) acting downstream of DMI2/SYMRK and LysM-RLKs, but independently of CCaMK or CYCLOPS, have been described in the angiosperm *M. truncatula* (30). In addition to these bifurcations in the CSP, the gain-of-function approach did not lead to the induction of the entire AMS transcriptomic response. It is likely that numerous genes under direct or indirect control of the CSP were not captured by the gain-of-function approach because of missing coregulators, such as the GRAS transcription factors NSP1, NSP2, or DELLA (31, 32). In addition, the CSP is under control of feedback loops that likely modulate the expression of the AMS-related genes, such as the phosphate starvation response *via* PHR1/2 (33, 34).

Phylogenomic analyses pointed out two directions that remain poorly explored. First, CCaMK, CYCLOPS, and a pro-ortholog of SYMRK are all present in streptophyte algae, thus predating the origin of AMS (35). Recent coexpression network analyses in the streptophyte alga *Zygnema circumcarinatum* indicate that the three genes may belong to separate pathways in algae (36). In that case, they would have assembled in a unified pathway after the divergence between Zygnematophyceae and land plants. Alternatively, the pathway was already formed, although each component was transcriptionally regulated in different ways as seen in extant Zygnematophyceae, and was directly recruited, exapted, as a whole during the evolution of AMS. Besides its origin and the recruitment for AMS, comparative phylogenomics indicated that, subsequently, the CSP may have been co-opted for other intracellular symbioses with diverse fungi and nitrogen-fixing bacteria (15). Genetics in legumes support this hypothesis for the root-nodule symbiosis (6). Here, we show that the CYCLOPS response element, *CYC-RE_pro_*, previously identified in the promoter of the root-nodule symbiosis-specific gene *NIN* (12, 19) is a reporter of CYCLOPS activation and of intracellular symbiosis outside of the root-nodule symbiosis context. Trait evolution is often mediated by the rewiring of existing gene regulatory networks, rewiring resulting from gene losses/gains, protein neofunctionalization, or gains and losses of *cis*-regulatory elements (37). Exploring further the rewiring of *cis-*regulatory elements downstream CCaMK-CYCLOPS that allowed plant lineages to transition from one symbiotic type to another represents the next challenge to be addressed.

Recent evo-devo studies conducted using *Marchantia polymorpha* by comparison with knowledge in angiosperms demonstrated that plant immunity mechanisms used by extant land plants were likely present in their most recent common ancestor. Indeed, reverse genetic approaches demonstrated that LysM-RLKs confer in both *M. polymorpha* and angiosperms the ability to perceive fungal chitin, and other microbe-derived polysaccharides, leading to the activation of plant defense responses (38). Downstream chitin perception, the PBL-RBOH cascade, described in angiosperms, is also essential to trigger defense responses such as ROS bursts in *M. polymorpha* (39). With the lack of very ancient fossils, reconstructing the morphology and biology of the first land plants is currently impossible. Despite this gap in our knowledge, the recent discoveries on plant immunity and the work presented here collectively indicate that 450 million years ago the first land plants were already able to regulate the interactions with their microbiota using specific signaling pathways.

## Material and Methods

### Phylogeny.

To reconstruct the phylogeny of CYCLOPS, we recovered protein sequences from a variety of land plants using hmmscan from HMMER v3.4 (40) with the HMM profile of the CYCLOPS domain (IPR040036). A set of 37 protein sequences was aligned using MAFFT v7.520 with the E-INS-i method (41). The phylogeny was reconstructed using IQ-TREE v2.2.2.3 with the model LG + C20 + F + G and support was provided with 1,000 ultrafast bootstrap replicates (42–44). The tree was rooted between bryophytes and vascular plants, and the tree and alignment were visualized using ETE3 v3.1.2 (45).

### Cloning.

The Golden Gate modular cloning system (46, 47) was used to prepare the plasmids as described by Rich et al. (2) for all constructs, except for *MpaSYMRK_pro_:GUS*. Levels 0, 1, and 2 used in this study are listed in Dataset S2 and held for distribution in the ENSA project core collection (https://www.ensa.ac.uk/). Sequences were domesticated (listed in Dataset S2), synthesized, and cloned into pMS (GeneArt, Thermo Fisher Scientific, Waltham).

Gateway system was used to construct *MpaSYMRK_pro_:GUS*. *MpaSYMRK_pro_* (2.4 kb) was amplified with 5’-GGGGACAAGTTTGTACAAAAAAGCAGGCTTCGCTTCTCAGAAACAACTCTA and 5’-GGGGAGCCACTTTGTACAAGAAAGCTGGGTCGTTCTGCTTCAAACCGAGAC and cloned by BP in pDOn207 and then into pMDC164 by LR (48).

#### Generation of CRISPR mutants in *M. paleacea* ssp. *paleacea*.

Constructs containing the *Arabidopsis thaliana* codon optimized Cas9 (49) under the *MpEF1a_pro_* promoter and two guide RNA under the *M. paleacea* or *M. polymorpha* U6*_pro_* promoter were transformed in *M. paleacea*. A total of nine alleles of *symrk* (*Marpal_utg000051g0090241*), thirteen of *ccamk* (*Marpal_utg000137g0173321*), and seven of *cyclops* (*Marpal_utg000051g0091871*) were genotyped and selected for phenotyping (SI Appendix, Table S2 and Dataset S2).

#### Generation of CRISPR mutants in *M. paleacea* ssp. *diptera*.

To generate mutants of CYCLOPS in *M. paleacea* ssp. *diptera* (*cyclops_4.1*, *cyclops_4.2*), plants were transformed with the construct containing *Arabidopsis*-codon-optimized Cas9 fused with *MpEF1a_pro_* promoter and one guide RNA (5’-GCTCGAACCATATTCATG) fused to the *MpU6-1_pro_*promoter. Two edited lines were selected for phenotyping.

### *Medicago* Assays.

Constructs were transformed in *Agrobacterium rhizogenes* A4TC24 by electroporation. Transformed strains were grown at 28 °C in Luria-Bertani medium supplemented with rifampicin and kanamycin (25 µg/mL). *M. truncatula* Jemalong A17 roots, were transformed with the different CYCLOPS-DD orthologs and the *CYC-RE_pro_::GUS* (Dataset S2) as described by Boisson-Dernier et al. (50), and grown on Fahraeus medium for 2 mo, selected with the DsRed marker present in all the constructs and GUS stained as in ref. 51. All transformation assays were performed independently on two batches of plants. Total number of plants observed are indicated in figures.

For nodulation and mycorrhization assays, *M. truncatula* plants with DsRed-positive roots were transferred to pots containing Zeolite substrate (50% fraction 1.0 to 2.5 mm, 50% fraction 0.5 to 1.0-mm, Symbiom). For nodulation assays, plants were watered with liquid Fahraeus medium (52). Wild-type *S. meliloti* RCR2011 pXLGD4 (GMI6526) was grown at 28 °C in tryptone yeast medium supplemented with 6 mM calcium chloride and 10 µg/mL tetracycline, rinsed with water, and diluted at OD600=0.02. Each pot was inoculated with 10 ml of bacterial suspension. For mycorrhization assays, each pot was inoculated with *ca*. 500 sterile spores of *Rhizophagus irregularis* DAOM 197198 provided by Agronutrition (Labège, France, https://www.agronutrition.com/en/contact-us/) and grown with a 16 h/8 h photoperiod at 22 °C/20 °C. Pots were watered once per week with Long Ashton medium containing 15 μM phosphate (53) (KNO_3_ 3.75 µM, Ca(NO_3_) 4H_2_O 4.75 µM, NaH_2_PO_4_ 2H_2_O 0.0117µM, MgSO_4_ 7H2O 2.5 µM)

### *Nissolia* Assays.

*N. brasiliensis* seeds provided by CIAT (Programade Recursos Geneticos, Valle, Colombia) were scarified with sulfuric acid for 5 min and surface-sterilized with bleach for 1 min. Seeds were washed 5 times with H_2_O at each step. Seeds were placed onto 0.8% (w/v) agar plates in a growth chamber (25 °C) under dark conditions for 3 d. Germinated seedlings were pierced with a needle that had been previously dipped in the *A. rhizogenes* inoculum at OD = 0.03, and placed on Fahraeus medium plates in a 25 °C growth chamber (16 h light/8 h dark). After 10 d, the untransformed roots (DSred-negative) were removed with a scalpel blade. After one month, transformed roots were screened for DsRed, and GUS-stained as indicated for *M. truncatula*.

### *Petunia* Assays.

#### *Petunia* genotyping.

*P. hybrida* LY3784 (*cyclops #1*) and 86-5 (*ccamk #1*) mutants were identified by searching a sequence-indexed dTph1 transposon database (53). Exact insert positions (expressed in base pairs downstream of the ATG start codon with the coding sequence as a reference) were determined by aligning the dTph1 flanking sequences with the genomic and cDNA sequences. All in silico identified candidate insertions were confirmed by PCR-based genotyping of the progeny from the selected insertion lines, using primers flanking the dTph1 transposon insertions (5’-ATGCAGCATAATATACCAGGAAATG and 5’-TGGGCTGGTTAGTAGTTTCAC for *CYCLOPS*, 5’-AAATTTTCCACACTCTTGATCAAACTC and 5’-AGCCACCTCTTCCAAGTATGTC for *CCaMK*). The following thermal profile was used for segregation analysis PCR: 10 cycles (94 °C for 15 s, 68 °C for 20 s minus 1 °C/cycle, 72 °C for 30 s), followed by 40 cycles (94 °C for 15 s, 58 °C for 20 s, and 72 °C for 30 s). The different insertion mutants were further systematically genotyped in subsequent crosses and segregation analyses. PCR products were analyzed by agarose gel electrophoresis. *Cyclops-1* has an insertion at 609 bp, *ccamk-1* at 262 bp.

#### Mycorrhization tests in *P. hybrida*.

Seeds were germinated by sowing in pots with wet soil, at the surface (without covering). Then, a minigreenhouse was placed over the pots to keep a high humidity and seeds were left to germinate in a growth chamber (25 °C day/22 °C night, 60% humidity, 16 h/8 h day/night). Germinated seedlings were transferred to zeolite (50% fraction 1.0 to 2.5 mm, 50% fraction 0.5 to 1.0-mm, Symbiom) soaked in Long-Ashton solution containing 15 μM of phosphate and inoculated with *ca.* 500 sterile spores/pot of *R. irregularis* DAOM 197198 provided by Agronutrition (Labège, France, https://www.agronutrition.com/en/contact-us/). Plants were grown in a growth chamber (25 °C day/22 °C night, 60% humidity, 16 h/8 h day/night) and watered regularly with the Long-Ashton solution. Root systems were harvested after 5 wk and stained with ink. Mycorrhization was quantified using the grid intersection method (54) in three independent assays.

### *Marchantia* Assays.

#### *M. paleacea* ssp*. paleacea* transformation.

Gemmae collected from axenic *M. paleacea* were grown in ½ strength Gamborg B5 media (G5768, Sigma, France) pH 5.7, 1.4% bacteriological agar (1330, Euromedex, France) for 4 to 5 wk.

For each construct, 15 to 25 gemmalings were blended for 15 s in a sterile, 250 ml stainless steel, bowl (Waring, USA) in 10 ml of 0M51C medium (KNO_3_ 2 g/L, NH_4_NO_3_ 0.4 g/L, MgSO_4_ 7H2O 0.37 g/L, CaCl_2_ 2H_2_O 0.3 g/L, KH_2_PO_4_ 0.275 g/L, L-glutamine 0.3 g/L, casamino-acids 1 g/L, Na_2_MoO_4_ 2H_2_O 0.25 mg/L, CuSO_4_ 5H2O 0.025 mg/L, CoCl_2_ 6H2O 0.025 mg/L, ZnSO_4_ 7H_2_O 2 mg/L, MnSO_4_ H_2_O 10 mg/L, H_3_BO_3_ 3 mg/L, KI 0.75 mg/L, EDTA ferric sodium 36.7 mg/L, myo-inositol 100 mg/L, nicotinic acid 1 mg/L, pyridoxine HCL 1 mg/L, thiamine HCL 10 mg/L). The blended plant tissues were transferred to 250 ml erlenmeyers containing 15 ml of 0M51C and kept at 20 °C, 16 h light/8 h dark, on a shaking table (200 RPM) for 3 d. Coculture was initiated by adding 100 µL of saturated *Agrobacterium tumefaciens* GV3101 liquid culture and acetosyringone (100 µM final). After 3 d, the plant fragments were washed by decantation three times with water, and plated on ½ Gamborg containing 200 mg/L amoxycilin (Levmentin, Laboratoires Delbert, FR) and 10 mg/L Hygromycin (Duchefa Biochimie, France).

For mutant complementations, gemmaes from selected mutant lines were surface sterilized in a bleach solution (~0.27%) for 75 s and washed in water twice before plating on ½ strength Gamborg B5 media. Transformation was performed as described above and selected on 500 nM chlorsulfuron (34322, Sigma).

#### *M. paleacea* ssp. *diptera* transformation.

Transformation was done as in ref. 4. Male parental line used to generate *cyclops* mutants was used as control in mycorrhization assays.

#### GUS-staining.

Plants, either mock-treated or inoculated with *R. irregularis* for 6 wk, were harvested. For staining, the GUS buffer is composed of phosphate buffer (0.1 M), EDTA (5 mM), K_3_Fe(CN)_6_ (0.5 mM), K_4_Fe(CN)6 (0.5 mM), X-Gluc (0.25 mg/ml, Euromedex, France), and H_2_O. After covering the plants with the GUS buffer, the tissues were incubated under vacuum for 5 min (twice), before incubating at 37 °C for 12 to 15 h. Several washes were performed with 70% ethanol to remove chlorophyll and clear the tissue. Tissues were stored in an aqueous solution containing EDTA (0.5 M).

#### Mycorrhization tests in *M. paleacea*.

Thalli of *M. paleacea* ssp. *paleacea* and ssp. *diptera* were grown on a zeolite substrate (50% fraction 1.0 to 2.5 mm, 50% fraction 0.5 to 1.0-mm, Symbiom) in 7 × 7 × 8 cm pots (five thalli per pot). Each pot was inoculated with *ca*. 1,000 sterile spores of *Rhizophagus irregularis* DAOM 197198 provided by Agronutrition (Labège, France, https://www.agronutrition.com/en/contact-us/) and grown with a 16 h/8 h photoperiod at 22 °C/20 °C. Pots were watered once a week with Long-Ashton medium containing 15 μM of phosphate. Five, six, or eight weeks postinoculation, thalli were cleared of chlorophyll using ethanol 100% for 24 h, then stored in an aqueous solution containing EDTA (0.5 mM). Cleared thalli were scanned, and the presence of the black/purple pigment indicative of colonization, scored as described in ref. 4. Colonization or lack of colonization was confirmed by ink staining and WGA-Alexa Fluor 488 staining on sections as presented below for the imaging and quantification.

#### Quantification of colonized *M. paleacea* rhizoids.

For each of the analyzed lines, six thalli were randomly picked. For each thallus, 8 to 10 longitudinal hand sections were made using a scalpel blade (~1 mm) for each thallus (taking one apex when multiple apexes were present), covering the entire area that has the potential to be colonized. The sections were cleared in 10% KOH for 14 h, rinsed three times with water and stained at room temperature for 30 min in 5% Sheaffer ink, 5% acetic acid. After staining, the sections were washed twice with water and left in the last wash for 2 h before counting. The number of colonized rhizoids was quantified under a stereomicroscope (Leica MZ75) for each section. The colonized rhizoids per thallus being the sum of colonized rhizoids for each section coming from that thallus. The experiment was repeated twice.

#### Microscopy on *M. paleacea*.

Thalli were embedded in 6% agarose and 100 µm transversal and horizontal sections were prepared using a Leica vt1000s vibratome. Sections were incubated two days in 10% KOH at 4 °C followed by water washes. The sections were then incubated in the staining solution, PBS with 1 µg/ml WGA-Alexa Fluor 488 (Invitrogen, France) overnight at 4 °C. GUS sections and *Marchantia* ssp. *diptera* ink staining (Fig. 1 and SI Appendix, Fig. S9) were acquired with a Zeiss Axiozoom V16 microscope. Other sections were imaged using a Nikon Ti Eclipse inverted microscope equipped with DS Ri2 camera and motorized XY stage. Large images of global sections are performed using the NIS AR 4.3 scan large image module that allow multifield acquisition and images stitching. These images were acquired with 10×/0.3 dry objective (0.73 pixel size) in brightfield and in fluorescence for WGA-alexa488 staining using a GFP band pass filter set (ex: 472/30 nm, em:520/35 nm). Close-up images were acquired using a Leica SP8 TCSPC confocal microscope and LAS X software with a 25× water immersion objective (Fluotar VISIR 25×/0.95 WATER) at zoom 5× (0.182 µm pixel size). WGA-alexa488 staining of fungi was excited with the 488 nm laser line and fluorescence was recovered between 500 nm and 550 nm. Brightfield image was also performed and merged. Images were processed with ImageJ.

### qRT-PCR.

RNAs of *M. paleacea cyclops* mutant lines or empty-vector control plants were extracted using a Direct-zol RNA MiniPrep Zymo kit according to the supplier’s recommendation on ~100 mg of ground frozen thalli.

Reverse transcription was performed using M-MLV (Promega) on 500 ng of RNA and qPCR was performed on 5× diluted cDNA in a BioRad CFX opus 384 thermocycler with SYBR Green (Sigma, France). Relative expression values were calculated using the reference genes *MpaEF1* and *MpaAPT2*. (data correspond to 2-ΔCT with ΔCT = CTgene of interest-CTaverageCTEF1 + APT2). (Table 1).

**Table 1.** List of primers used for qRT-PCR

Primer	Sequence
MpaEF1 qPCR F	5’-AATGTGTTGAGCAGCTTGGC
MpaEF1 qPCR R	5’-ACGTTCCAAGTACTCTCGAGC
MpaAPT2 qPCR F	5’-GGGTACACTTGCTGCAGGAA
MpaAPT2 qPCR R	5’-CTCACGGCCCTTTAGATCCG
MpaSYMPT qPCR F	5’-ACGGCAAGCAAGATCATGGA
MpaSYMPT qPCR R	5’-GGACCAGGAACGTGAAGAGG
MpaSTR qPCR F	5’-TCGTCTCTCATCACCACCAA
MpaSTR qPCR R	5’-ATCCGCATGTCAAGAAGGAC
RiTEF qPCR F	5’-GCCATACCGCTCATATTGCT
RiTEF qPCR R	5’-CTCAACACACATCGGTTTGG

### RNA-seq.

#### Library preparation.

Three independent lines expressing *MpEF1a_pro_:MtCYCLOPS-DD*, *MpEF1a_pro_:MtCCaMK-Kin*, *MpEF1a_pro_:MpaCYCLOPS-DD* or transformed with an empty vector control (Line 132, control line) were harvested five weeks after transfer to zeolite substrate (50% fraction 1.0 to 2.5 mm, 50% fraction 0.5 to 1.0-mm, Symbiom, Czech Republik) in 7 × 7 × 8 cm pots (five thalli per pot). Thalli from each pot were pooled in a single sample, flash-frozen and stored at −70 °C until extraction. TRI-reagent (Sigma, France) extraction was performed according to the supplier's recommendation on ~100 mg of ground frozen thalli. Around 2 µg of RNA was treated with RQ1 DNase (Promega) and sent for sequencing to Genewiz/Azenta (Leipzig, Germany). Illumina libraries were prepared with the NEBnext ultra II RNA directional kit and sequenced on a NovaSeq platform.

#### Differential gene expression analysis.

All sequenced RNA-seq libraries were mapped against the reference genome of *M. paleacea* (2) using nextflow (55) (v21.04.1, build 5556) run nf-core/rnaseq (56, 57) (v3.4) using *-profile debug, genotoul --remove_ribo_rna --skip_qc --aligner star_salmon* options. The workflow used bedtools (58) (v2.30.0), bioconductor-summarized experiment (v1.20.0), bioconductor-tximeta (v1.8.0), gffread (59) (v0.12.1), picard (v2.25.7), salmon (60) (v1.5.2), samtools (61) (v1.13), star (62) (v2.6.1d), stringtie (63) (v2.1.7), Trimgalore (v0.6.7, GitHub—FelixKrueger/TrimGalore: A wrapper around Cutadapt and FastQC to consistently apply adapter and quality trimming to FastQ files, with extra functionality for RRBS data), cutadapt (64) (v3.4) and ucsc (v377). Differentially expressed genes (DEGs) for the different lines were estimated using “*edgeR*” (65) in R (v4.1.2, R Core Team 2021). Briefly, low expressed genes with less than 10 reads across each class of samples were removed. Then, gene counts were normalized by library size and trimmed mean of M-values (*i.e.* TMM) normalization method (66). We estimated differentially expressed genes (DEGs) by comparing each transformed genotype (*MpEF1a_pro_:MtCYCLOPS-DD*, *MpEF1a_pro_:MtCCaMK-Kin,* and *MpEF1a_pro_:MpaCYCLOPS-DD*) to empty vector plants. Genes were considered differentially expressed when the FDR was below 0.05 (Benjamini–Hochberg correction).

The code used is available here https://figshare.com/s/eac7dfa06815d7c4f665.

#### Multidimensional scaling (MDS) plot.

In order to define the similarity in the transcriptomic signatures between the overexpressing lines we conducted a multidimensional scaling (MDS) plot using glMDSPlot function of the glimma (65, 67) package v2.12.0. The expression data were normalized by library size and the TMM method provided in the edgeR [2] package v4.0.2.

#### Statistical analyses.

To estimate if the observed overlap between genes deregulated by the overexpression of the different constructs in *M. paleacea* (*MpEF1a_pro_:MtCYCLOPS-DD*, *MpEF1a_pro_:MtCCaMK-Kin,* and *MpEF1a_pro_: MpaCYCLOPS-DD*) differed from random expectations, we randomly sampled (10,000 times) the same number of genes than the number of genes deregulated in each treatment, and compared these random datasets to estimate the random overlap. Quantile metrics were computed for each comparison. The code used is available here https://figshare.com/s/eac7dfa06815d7c4f665.

To estimate the differences between colonization rates quantified by the presence of AMS-induced pigment in *M. paleacea* apexes, we used a pairwise comparison of proportions (Chi2) to the control line and a BH p-value adjustment.

qRT-PCR results were compared to the control lines using a Student’s *t* test.

## Supplementary Material

Appendix 01 (PDF)

Dataset S01 (XLSX)

Dataset S02 (XLSX)

## Data Availability

RNA-seq; R scripts developed to analyse RNA-seq data have been deposited in NCBI; Figshare (PRJNA1051818 (68)). Some study data available (Plasmids and transgenic lines generated in this study) are available upon request. For the transfer of transgenic material, appropriate information on import permits will be required from the receiver. RNA-seq reads were deposited on the SRA with the Bioproject number PRJNA1051818. R scripts developed to analyze RNA-seq data are available under the FigShare project page https://figshare.com/s/eac7dfa06815d7c4f665 (68). Any additional information required to reanalyze the data reported in this paper is available from the lead contacts (pierre-marc.delaux@cnrs.fr or tatiana.vernie@univ-tlse3.fr) upon request.

## References

[1] D. J. Beerling, The Emerald Planet: How Plants Changed Earth’s History (Oxford University Press, 2008).

[2] M. K. Rich , Lipid exchanges drove the evolution of mutualism during plant terrestrialization. Science **372**, 864–868 (2021). 34016782 10.1126/science.abg0929

[3] K. A. Pirozynski, D. W. Malloch, The origin of land plants: A matter of mycotrophism. BioSystems **6**, 153–164 (1975). 1120179 10.1016/0303-2647(75)90023-4

[4] K. Kodama , An ancestral function of strigolactones as symbiotic rhizosphere signals. Nat. Commun. **13**, 3974 (2022). 35803942 10.1038/s41467-022-31708-3 PMC9270392

[5] P.-M. Delaux , Reconstructing trait evolution in plant evo–devo studies. Curr. Biol. **29**, R1110–R1118 (2019). 31689391 10.1016/j.cub.2019.09.044

[6] M. Parniske, Arbuscular mycorrhizae: The mother of plant root endosymbioses. Nat. Rev. Microbiol. **6**, 763–775 (2008). 18794914 10.1038/nrmicro1987

[7] G. Endre , A receptor kinase gene regulating symbiotic nodule development. Nature **417**, 962–966 (2002). 12087406 10.1038/nature00842

[8] S. Stracke , A plant receptor-like kinase required for both bacterial and fungal symbiosis. Nature **417**, 959–962 (2002). 12087405 10.1038/nature00841

[9] J. Levy , A putative Ca2+ and Calmodulin-dependent protein kinase required for bacterial and fungal symbioses. Science (80-). **303**, 1361–1364 (2004). 10.1126/science.1093038 14963335

[10] K. Yano , CYCLOPS, a mediator of symbiotic intracellular accommodation. Proc. Natl. Acad. Sci. **105**, 20540–20545 (2008). 19074278 10.1073/pnas.0806858105 PMC2629324

[11] E. Messinese , A novel nuclear protein interacts with the symbiotic DMI3 calcium- and calmodulin-dependent protein kinase of Medicago truncatula. Mol. Plant. Microbe. Interact. **20**, 912–921 (2007). 17722695 10.1094/MPMI-20-8-0912

[12] S. Singh, K. Katzer, J. Lambert, M. Cerri, M. Parniske, CYCLOPS, A DNA-binding transcriptional activator, orchestrates symbiotic root nodule development. Cell Host Microbe **15**, 139–152 (2014). 24528861 10.1016/j.chom.2014.01.011

[13] C. Kistner, M. Parniske, Evolution of signal transduction in intracellular symbiosis. Trends Plant Sci. **7**, 511–518 (2002). 12417152 10.1016/s1360-1385(02)02356-7

[14] P. M. Delaux , Comparative phylogenomics uncovers the impact of symbiotic associations on host genome evolution. PLoS Genet. **10**, e1004487 (2014). 25032823 10.1371/journal.pgen.1004487 PMC4102449

[15] G. V. Radhakrishnan , An ancestral signalling pathway is conserved in intracellular symbioses-forming plant lineages. Nat. Plants **6**, 280–289 (2020), 10.1038/s41477-020-0613-7. 32123350

[16] P. Pimprikar , A CCaMK-CYCLOPS-DELLA complex activates transcription of RAM1 to regulate arbuscule branching. Curr. Biol. **26**, 987–998 (2016). 27020747 10.1016/j.cub.2016.01.069

[17] M. R. Cerri , The ERN1 transcription factor gene is a target of the CCaMK/CYCLOPS complex and controls rhizobial infection in Lotus japonicus. New Phytol. **215**, 323–337 (2017). 28503742 10.1111/nph.14547

[18] X. Gong, E. Jensen, S. Bucerius, M. Parniske, A CCaMK/Cyclops response element in the promoter of Lotus japonicus calcium-binding protein 1 (CBP1) mediates transcriptional activation in root symbioses. New Phytol. **235**, 1196–1211 (2022). 35318667 10.1111/nph.18112

[19] C. Cathebras , A novel cis-element enabled bacterial uptake by plant cells. bioRxiv [Preprint] (2022). 10.1101/2022.03.28.486070 (Accessed 16 December 2024). PMC12830364 41482520

[20] M. Griesmann , Phylogenomics reveals multiple losses of nitrogen-fixing root nodule symbiosis. Science **361**, eaat1743 (2018), 10.1126/science.aat1743. 29794220

[21] C. P. Humphreys , Mutualistic mycorrhiza-like symbiosis in the most ancient group of land plants. Nat. Commun. **1**, 103 (2010). 21045821 10.1038/ncomms1105

[22] M. N. Puttick , The interrelationships of land plants and the nature of the ancestral embryophyte. Curr. Biol. **28**, 733–745.e2 (2018). 29456145 10.1016/j.cub.2018.01.063

[23] X. R. Li , Nutrient regulation of lipochitooligosaccharide recognition in plants via NSP1 and NSP2. Nat. Commun. **13** (2022). 10.1038/s41467-022-33908-3 PMC9616857 36307431

[24] K. Miyata , OsSYMRK plays an essential role in AM symbiosis in rice (Oryza sativa). Plant Cell Physiol. **64**, 378–391 (2023). 36688592 10.1093/pcp/pcad006

[25] Y. Jin , IPD3 and IPD3L function redundantly in rhizobial and mycorrhizal symbioses. Front. Plant Sci. **9**, 267 (2018), 10.3389/fpls.2018.00267. 29616050 PMC5865340

[26] B. Wang , Presence of three mycorrhizal genes in the common ancestor of land plants suggests a key role of mycorrhizas in the colonization of land by plants. New Phytol. **186**, 514–525 (2010). 20059702 10.1111/j.1469-8137.2009.03137.x

[27] C. Gleason , Nodulation independent of rhizobia induced by a calcium-activated kinase lacking autoinhibition. Nature **441**, 1149–1152 (2006). 16810256 10.1038/nature04812

[28] L. Xue , Network of GRAS transcription factors involved in the control of arbuscule development in *Lotus japonicus*. Plant Physiol. **167**, 854–871 (2015). 25560877 10.1104/pp.114.255430 PMC4348782

[29] A. H. C. Lam, A. Cooke, H. Wright, D. M. Lawson, M. Charpentier, Evolution of endosymbiosis-mediated nuclear calcium signaling in land plants. Curr. Biol. **34**, 2212–2220.e7 (2024). 38642549 10.1016/j.cub.2024.03.063

[30] S. Ivanov, M. J. Harrison, Receptor-associated kinases control the lipid provisioning program in plant-fungal symbiosis. Science **383**, 443–448 (2024). 38271524 10.1126/science.ade1124

[31] C. Fonouni-Farde , DELLA-mediated gibberellin signalling regulates Nod factor signalling and rhizobial infection. Nat. Commun. **7**, 12636 (2016). 27586842 10.1038/ncomms12636 PMC5025792

[32] Y. Jin , DELLA proteins are common components of symbiotic rhizobial and mycorrhizal signalling pathways. Nat. Commun. **7**, 12433 (2016). 27514472 10.1038/ncomms12433 PMC4990646

[33] J. Shi , A phosphate starvation response-centered network regulates mycorrhizal symbiosis. Cell **184**, 5527–5540.e18 (2021). 34644527 10.1016/j.cell.2021.09.030

[34] D. Das , Phosphate starvation response transcription factors enable arbuscular mycorrhiza symbiosis. Nat. Commun. **13**, 477 (2022). 35078978 10.1038/s41467-022-27976-8 PMC8789775

[35] P.-M. Delaux , Algal ancestor of land plants was preadapted for symbiosis. Proc. Natl. Acad. Sci. **112**, 13390–13395 (2015). 26438870 10.1073/pnas.1515426112 PMC4629359

[36] X. Feng , Genomes of multicellular algal sisters to land plants illuminate signaling network evolution. Nat. Genet. **56**, 1018–1031 (2024). 38693345 10.1038/s41588-024-01737-3 PMC11096116

[37] S. B. Carroll, Evo-devo and an expanding evolutionary synthesis: A genetic theory of morphological evolution. Cell **134**, 25–36 (2008). 18614008 10.1016/j.cell.2008.06.030

[38] I. Yotsui , LysM-mediated signaling in Marchantia polymorpha highlights the conservation of pattern-triggered immunity in land plants. Curr. Biol. **33**, 3732–3746.e8 (2023). 37619565 10.1016/j.cub.2023.07.068

[39] J. Chu , Conservation of the PBL-RBOH immune module in land plants. Curr. Biol. **33**, 1130–1137.e5 (2023). 36796360 10.1016/j.cub.2023.01.050

[40] S. R. Eddy, A new generation of homology search tools based on probabilistic inference. Genome Inform. **23**, 205–211 (2009). 20180275

[41] K. Katoh, D. M. Standley, MAFFT multiple sequence alignment software version 7: Improvements in performance and usability. Mol. Biol. Evol. **30**, 772–780 (2013), 10.1093/molbev/mst010. 23329690 PMC3603318

[42] D. T. Hoang, O. Chernomor, A. Von Haeseler, B. Q. Minh, L. S. Vinh, UFBoot2: Improving the ultrafast bootstrap approximation. Mol. Biol. Evol. **35**, 518–522 (2018). 29077904 10.1093/molbev/msx281 PMC5850222

[43] L. S. Quang, O. Gascuel, N. Lartillot, Empirical profile mixture models for phylogenetic reconstruction. Bioinformatics **24**, 2317–2323 (2008). 18718941 10.1093/bioinformatics/btn445

[44] B. Q. Minh , IQ-TREE 2: New models and efficient methods for phylogenetic inference in the genomic era. Mol. Biol. Evol. **37**, 1530–1534 (2020). 32011700 10.1093/molbev/msaa015 PMC7182206

[45] S. Q. Le, O. Gascuel, An improved general amino acid replacement matrix. Mol. Biol. Evol. **25**, 1307–1320 (2008). 18367465 10.1093/molbev/msn067

[46] C. Engler , A golden gate modular cloning toolbox for plants. ACS Synth. Biol. **3**, 839–843 (2014). 24933124 10.1021/sb4001504

[47] N. J. Patron , Standards for plant synthetic biology: A common syntax for exchange of DNA parts. New Phytol. **208**, 13–19 (2015). 26171760 10.1111/nph.13532

[48] M. D. Curtis, U. Grossniklaus, A gateway cloning vector set for high-throughput functional analysis of genes in planta. Plant Physiol. **133**, 462–469 (2003). 14555774 10.1104/pp.103.027979 PMC523872

[49] J. F. Li , Multiplex and homologous recombination-mediated genome editing in Arabidopsis and Nicotiana benthamiana using guide RNA and Cas9. Nat. Biotechnol. **31**, 688–691 (2013). 23929339 10.1038/nbt.2654 PMC4078740

[50] A. Boisson-Dernier , *Agrobacterium rhizogenes—*Transformed roots of *Medicago truncatula* for the study of nitrogen-fixing and endomycorrhizal symbiotic associations. Mol. Plant-Microbe Interact. **14**, 695–700 (2001). 11386364 10.1094/MPMI.2001.14.6.695

[51] T. Vernié , The NIN transcription factor coordinates diverse nodulation programs in different tissues of the *Medicago truncatula* Root. Plant Cell **27**, 3410–3424 (2015). 26672071 10.1105/tpc.15.00461 PMC4707452

[52] R. Catoira , Four genes of Medicago truncatula controlling components of a Nod factor transduction pathway. Plant Cell **12**, 1647–1666 (2000), 10.1105/tpc.12.9.1647. 11006338 PMC149076

[53] C. Balzergue, V. Puech-Pags, G. Bécard, S. F. Rochange, The regulation of arbuscular mycorrhizal symbiosis by phosphate in pea involves early and systemic signalling events. J. Exp. Bot. **62**, 1049–1060 (2011). 21045005 10.1093/jxb/erq335 PMC3022399

[54] M. Giovannetti, B. Mosse, An evaluation of techniques for measuring vesicular arbuscular mycorrhizal infection in roots. New Phytol. **84**, 489–500 (1980).

[55] P. di Tommaso , Nextflow enables reproducible computational workflows. Nat. Biotechnol. **35**, 316–319 (2017). 28398311 10.1038/nbt.3820

[56] P. A. Ewels , The nf-core framework for community-curated bioinformatics pipelines. Nat. Biotechnol. **38**, 276–278 (2020). 32055031 10.1038/s41587-020-0439-x

[57] nf-core/rnaseq: nf-core/rnaseq v3.12.0–Osmium Octopus. 10.5281/ZENODO.7998767.

[58] A. R. Quinlan, I. M. Hall, BEDTools: A flexible suite of utilities for comparing genomic features. Bioinformatics **26**, 841–842 (2010). 20110278 10.1093/bioinformatics/btq033 PMC2832824

[59] G. Pertea, M. Pertea, GFF Utilities: GffRead and GffCompare. F1000Research **9**, 304 (2020). 10.12688/f1000research.23297.1 PMC7222033 32489650

[60] R. Patro, G. Duggal, M. I. Love, R. A. Irizarry, C. Kingsford, Salmon provides fast and bias-aware quantification of transcript expression. Nat. Methods **14**, 417–419 (2017). 28263959 10.1038/nmeth.4197 PMC5600148

[61] H. Li , The sequence alignment/map format and SAMtools. Bioinformatics **25**, 2078–2079 (2009). 19505943 10.1093/bioinformatics/btp352 PMC2723002

[62] A. Dobin , STAR: Ultrafast universal RNA-seq aligner. Bioinformatics **29**, 15–21 (2013). 23104886 10.1093/bioinformatics/bts635 PMC3530905

[63] M. Pertea , StringTie enables improved reconstruction of a transcriptome from RNA-seq reads. Nat. Biotechnol. **33**, 290–295 (2015). 25690850 10.1038/nbt.3122 PMC4643835

[64] M. Martin, Cutadapt removes adapter sequences from high-throughput sequencing reads. EMBnet.journal **17**, 10–12 (2011).

[65] M. D. Robinson, D. J. McCarthy, G. K. Smyth, edgeR: A Bioconductor package for differential expression analysis of digital gene expression data. Bioinformatics **26**, 139–140 (2010). 19910308 10.1093/bioinformatics/btp616 PMC2796818

[66] M. D. Robinson, A. Oshlack, A scaling normalization method for differential expression analysis of RNA-seq data. Genome Biol. **11**, R25 (2010). 20196867 10.1186/gb-2010-11-3-r25 PMC2864565

[67] S. Su , Glimma: Interactive graphics for gene expression analysis. Bioinformatics **33**, 2050–2052 (2017). 28203714 10.1093/bioinformatics/btx094 PMC5870845

[68] T. Vernie, C. Libourel, P.-M. Delaux, Overexpression of the common symbiosis pathway in Marchantia paleacea and Marchantia polymorpha. NCBI. https://www.ncbi.nlm.nih.gov/bioproject/1051818. Deposited 13 December 2024.

