# Supplementary material for: Conservation of symbiotic signaling since the most recent common ancestor of land plants
Source: Proc Natl Acad Sci U S A. 2024 Dec 31;122(1):e2408539121. doi: 10.1073/pnas.2408539121 (PMC11725925; doi:10.1073/pnas.2408539121)
Supplement: Supplementary file 1 — Appendix 01 (PDF) [file pnas.2408539121.sapp.pdf]

PNAS

Supporting Information for

Conservation of symbiotic signalling since the most recent common ancestor of land plants

Tatiana Vernié[1]*, Mélanie Rich[1], Tifenn Pellen[1], Eve Teyssier[1], Vincent Garrigues[1], Lucie Chauderon[1], Lauréna Medioni[1], Fabian van Beveren[1], Cyril Libourel[1], Jean Keller[1], Camille Girou[1], Corinne Lefort[1], Aurélie Le Ru[2], Yves Martinez[2], Didier Reinhardt[3], Kyoichi Kodama[4], Syota Shimazaki[4], Patrice Morel[5], Junko Kyozuka[4], Malick Mbengue[1], Michiel Vandenbussche[5], Pierre-Marc Delaux[1]*

[1] Laboratoire de Recherche en Sciences Végétales (LRSV), Université de Toulouse, CNRS, UPS, INP Toulouse, Castanet-Tolosan, France.

[2] Fédération de Recherche 3450, Plateforme Imagerie, Pôle de Biotechnologie Végétale, 31326 Castanet-Tolosan, France.

[3] Department of Biology, University of Fribourg, Rte Albert-Gockel 3, 1700, Fribourg, Switzerland.

[4] Graduate School of Life Sciences, Tohoku University, Sendai, Japan.

[5] Laboratoire Reproduction et Développement des Plantes, Univ Lyon, ENS de Lyon, UCB Lyon 1, CNRS, INRA, Lyon, France.

*Correspondence: tatiana.vernie@univ-tlse3.fr, pierre-marc.delaux@cnrs.fr

This PDF file includes:

Figures S1 to S9
Tables S1 to S3
Legends for Datasets S1 to S2

Other supporting materials for this manuscript include the following:

Datasets S1 to S2

Supplementary Figure 1: *CYC_RE*$_{pro}$ expression in *M. truncatula* roots

A. The *CYC-RE*$_{pro}$*:GUS* signal in *M. truncatula* roots inoculated with *S. meliloti* is localized in young dividing cells (triangle) at the origin of nodule primordium, in young nodule (star) and at the base of developing and 12 days-old nodules (arrow). **B-G.** *M. truncatula* roots expressing *CYC-RE*$_{pro}$*:GUS* six weeks post inoculation with *R. irregularis* (**B, D, F**) or non-inoculated (**C, E, G**). In inoculated roots the blue signal is stronger and associated with colonized cells, whereas in non inoculated roots, only some weak and diffuse signal can be observed in a few roots. In **F**, an overlay with WGA-Alexa Fluor 488 signal associated with *R. irregularis* is shown. Scale bars correspond to 1 cm in **B** and **C**, and to 100µm in other panels. **G-H.** *N. brasiliensis* roots transformed with *CYC-RE*$_{pro}$*:GUS* (**H**) and *CYC-RE*$_{pro}$*:GUS* + *LjUBI*$_{pro}$*:MtCYCLOPS DD* (**I**). Number of plants showing a similar expression pattern out of the total number of transformed plants is indicated.

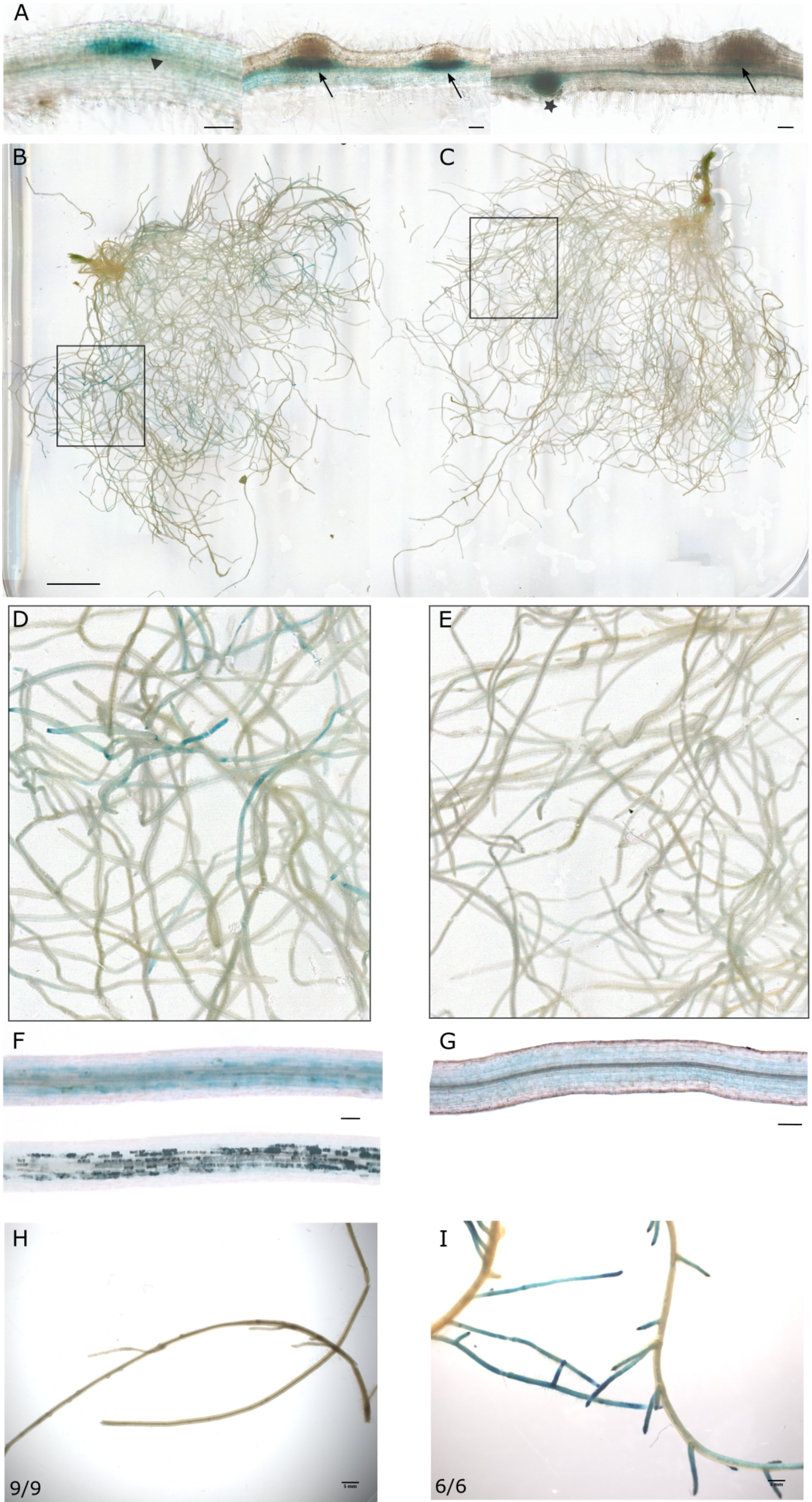

Supplemental Figure 2: CDS and protein sequence alignments of MpaSYMRK in wild type and *M. paleacea* mutant lines.

A and B. Genomic alignment of the two first exons of *MpaSYMRK*. Pairs of sgRNAs used for CRISPR/Cas9 are underlined in different colors. Premature stop codons are underlined in black.

C. Protein sequence alignment of the two first exons of MpaSYMRK from the different mutant lines.

Alignments were performed using Clustal Omega and visualized in AliView.

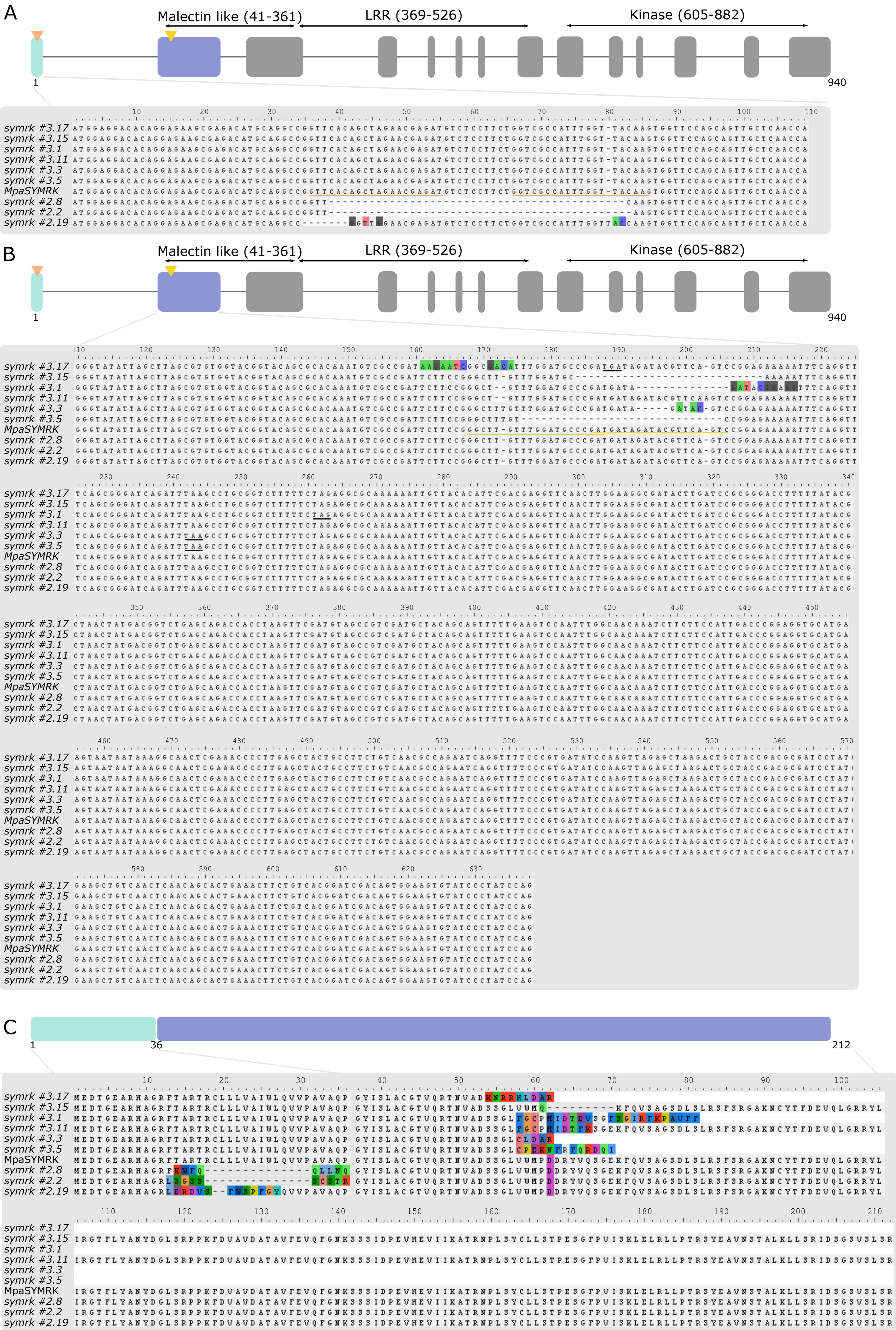

Supplemental Figure 3: Symbiotic phenotypes of the *Marchantia paleacea symrk* mutants.

A. Exons/introns structure of the *MpaSYMRK* genomic sequence. The number of amino acids and predicted domains are indicated. CRISPR/Cas9 was conducted on exon 1 and 2 (arrowheads indicate position of sgRNAs).

B. Colonization rates of *M. paleacea symrk* mutant lines at six weeks post inoculation with *R. irregularis*. Colonization rates are estimated by quantifying the presence of AMS-induced pigment in *M. paleacea* apexes. Predicted protein structures are indicated on the left for the two first exons. Red asterisks indicate the presence of a premature stop codon in the mutant line. *** = statistical difference (p<0,001) calculated with a pairwise comparison of proportions (Chi2) to the control line and a BH p-value adjustment. One out of two independent assays is shown, numbers of observed thalli in the assay are indicated (n).

C. Transversal sections of *symrk* and control lines six weeks post inoculation with *R. irregularis*. *R. irregularis* is visualized with WGA-Alexa Fluor 488. For each line, bright field, Alexa Fluor 488 and overlay are shown and presence of the fungi was checked at cellular level by confocal microscopy (Close-up pictures Alexa Fluor 488 and overlays). Scale bar=100µm (Nikon microscopy) and 10µm (confocal microscopy).

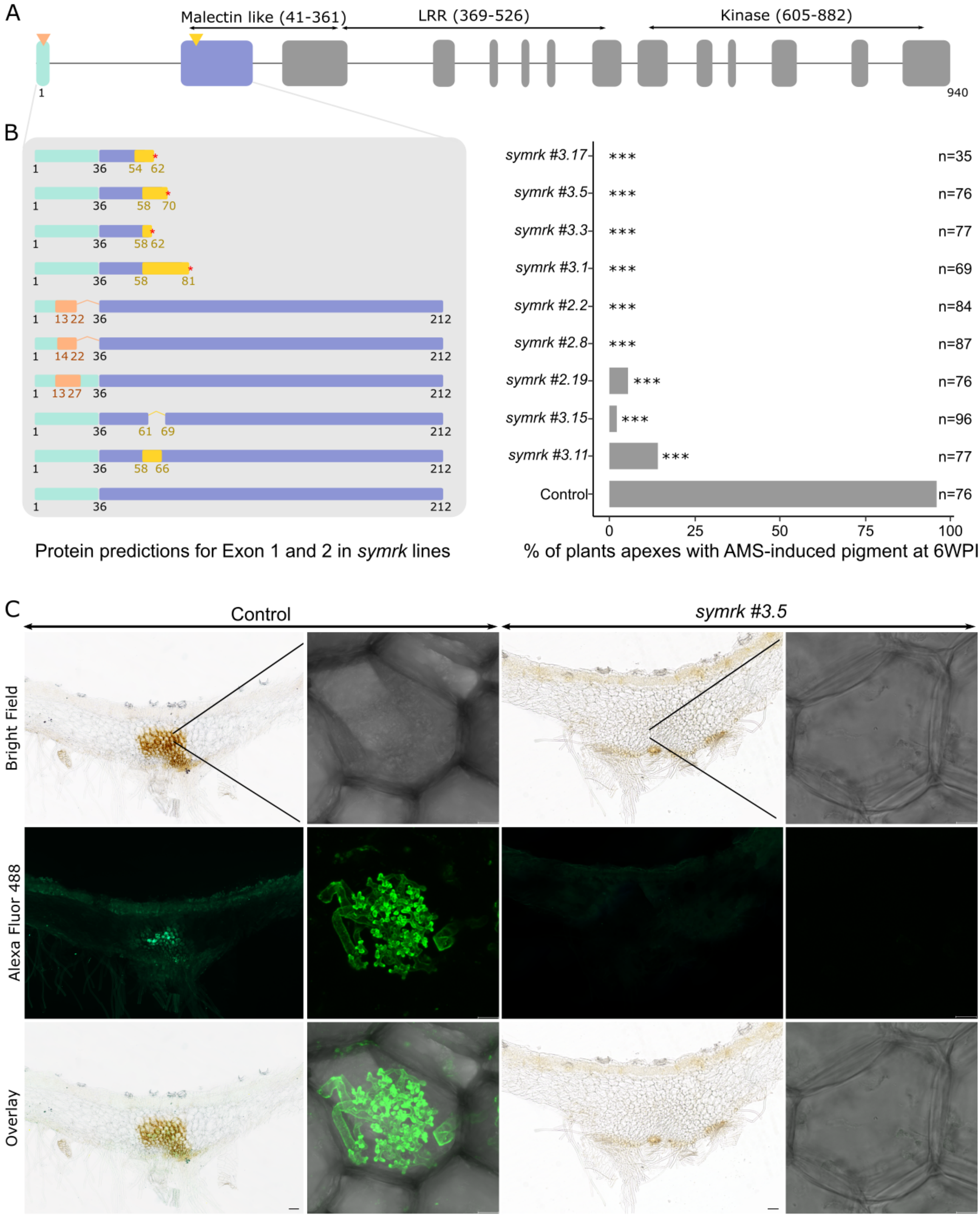

Supplemental Figure 4: CDS and protein sequence alignments of MpaCCaMK in wild type and *M. paleacea* mutant lines.
A. Genomic alignment of the first exon of *MpaCCaMK*. Pairs of sgRNAs used for CRISPR/Cas9 are underlined in different colors. Premature stop codons are underlined in black.
B. Protein sequence alignment of the first exon of MpaCCaMK from the different mutant lines.
Alignments were done using Clustal Omega and visualized in AliView.

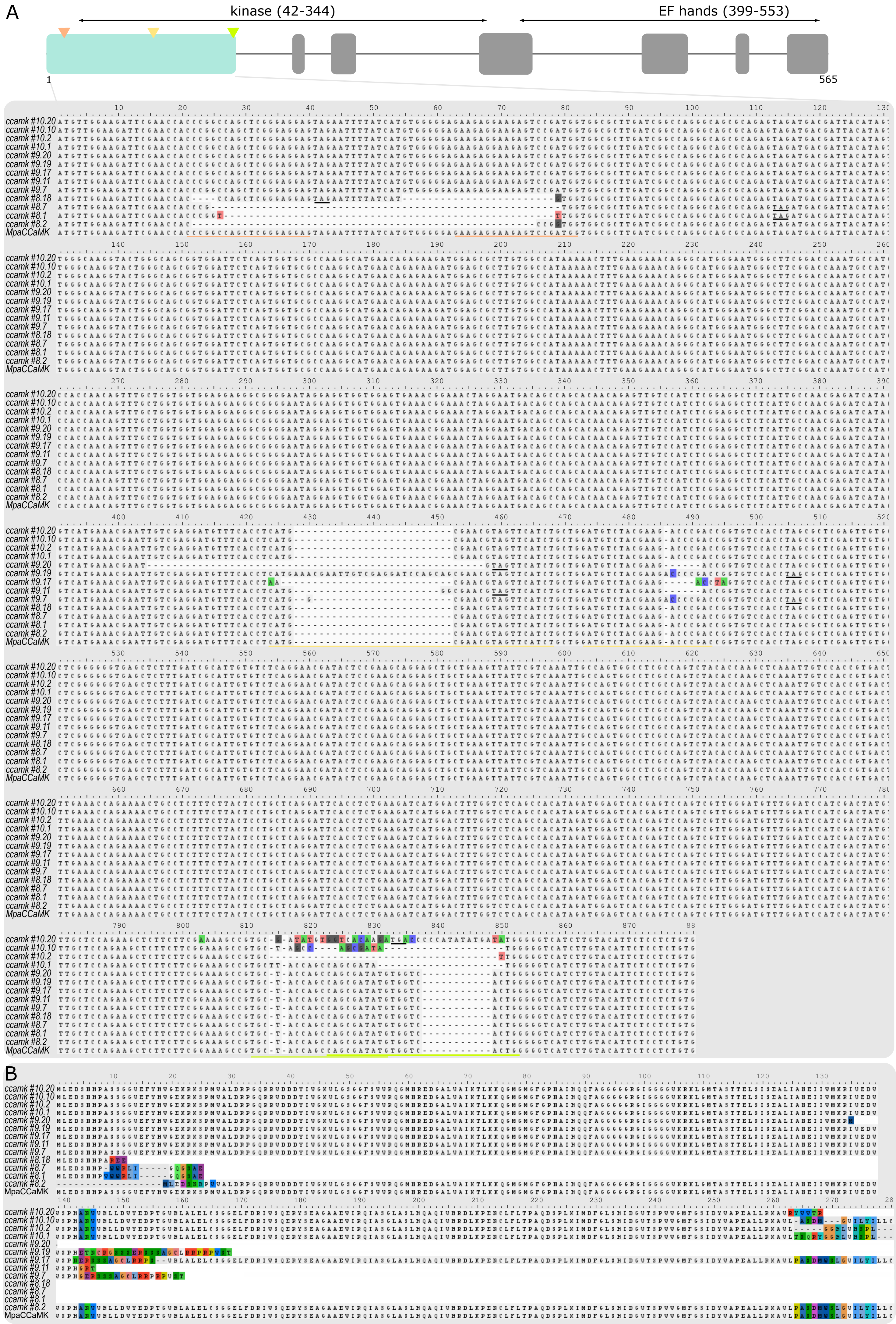

Supplemental Figure 5: Genomic and proteic alignments of MpaCYCLOPS in wild type and *M. paleacea ssp paleacea* mutant lines.

A. Genomic alignment of the first exon of *MpaCYCLOPS*. Pairs of sgRNAs used for CRISPR/Cas9 are underlined in different colors. Premature stop codons are underlined in black and the predicted new in-frame start codons are underlined in blue. The N-terminal domain absent from angiosperms is indicated in green, the regulatory domain in black.

B. Protein sequence alignment of the first exon of MpaCYCLOPS until premature stop codon if a stop codon is present.

C. Protein sequence alignment of the first exon of MpaCYCLOPS from sequences with new predicted in-frame start codons in the different mutant lines. Alignments were performed using Clustal Omega and visualized in AliView.

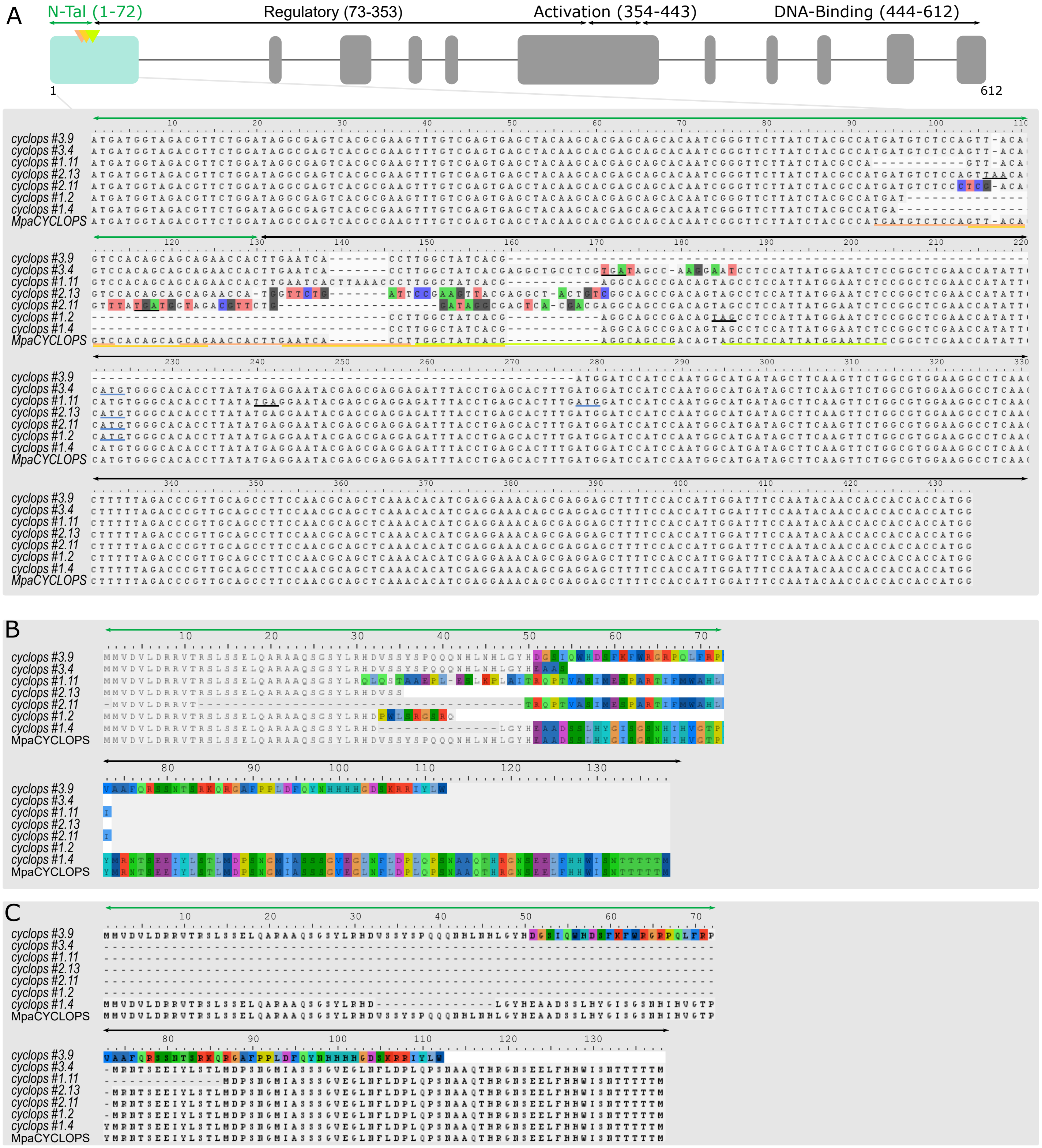

Supplemental Figure 6: Symbiotic phenotypes of the *Marchantia paleacea ccamk* mutants.
A. Exons/introns structure of the *MpaCCaMK* genomic sequence. The number of amino acids and predicted domains are indicated. CRISPR/Cas9 was conducted on exon 1 (arrowheads indicate position of sgRNAs).
B. Colonization rates of *M. paleacea ccamk* lines at six weeks post inoculation with *R. irregularis.* Colonization rates are estimated by quantifying the presence of AMS-induced pigment in *M. paleacea* apexes. Predicted protein structures are indicated on the left for the first targeted exon. Red asterisks indicate the presence of a premature stop codon in the mutant line. *** = statistical difference (p<0,001) calculated with a pairwise comparison of proportions (Chi2) to the control line and a BH p-value adjustment. One out of three independent assays is shown, numbers of observed thalli in the assay are indicated (n).
C. Transversal sections of *ccamk* and control lines six weeks post inoculation with *R. irregularis*. *R. irregularis* is visualized with WGA-Alexa Fluor 488. For each line, bright field, Alexa Fluor 488 and overlay are shown and presence of the fungi was checked at cellular level by confocal microscopy (Close-up pictures Alexa Fluor 488 and overlays). Scale bar=100µm (Nikon microscopy) and 10µm (confocal microscopy).

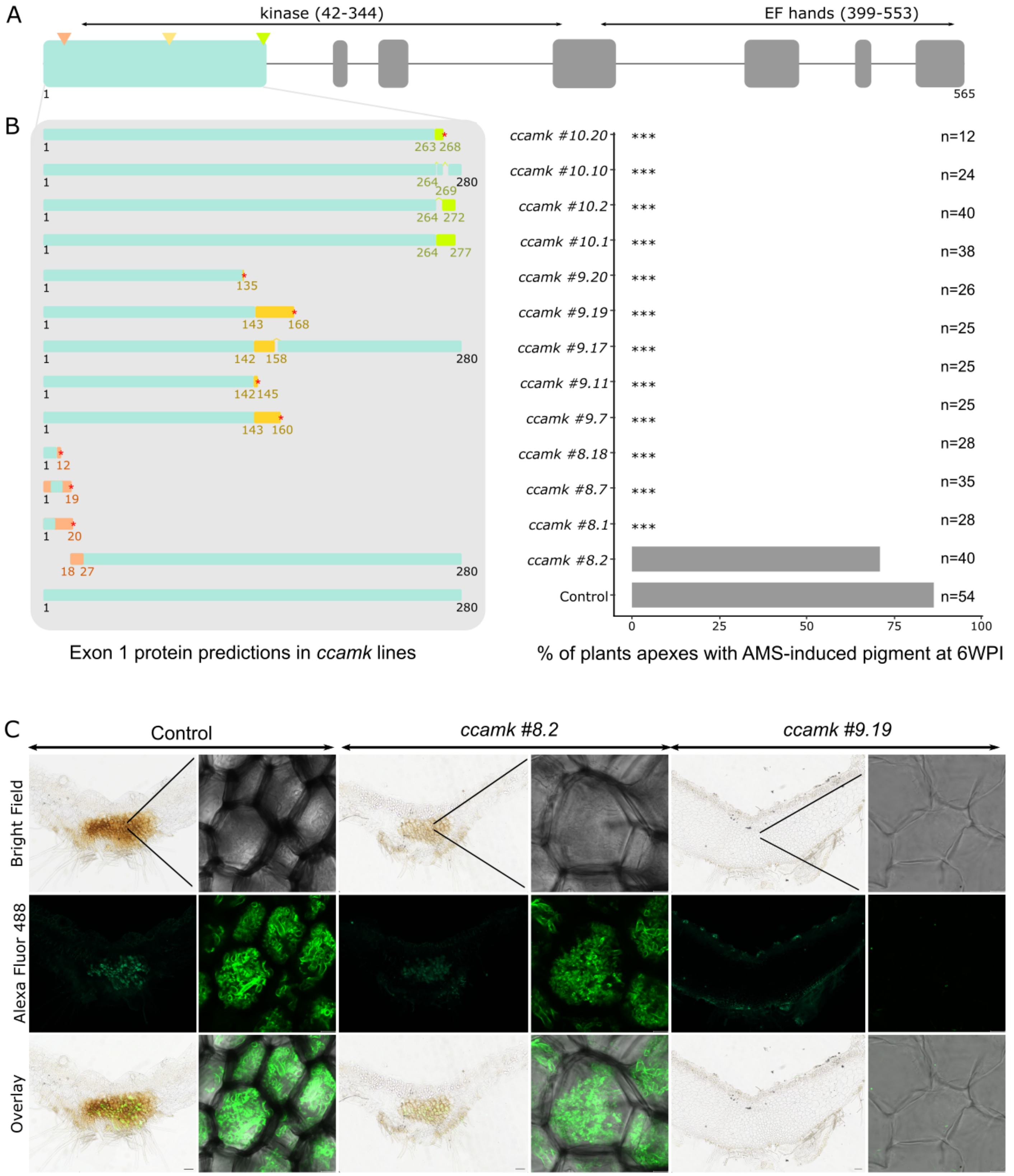

Supplemental Figure 7: Symbiotic phenotypes of the *Marchantia paleacea cyclops* mutants.

A. Exons/intron structure of the *MpaCYCLOPS* genomic sequence. Number of amino acids and predicted domains are indicated. CRISPR/Cas9 was conducted on exon 1 (arrowheads indicate position of sgRNAs).

B. Colonization rates of *M. paleacea cyclops* lines at five and eight weeks post inoculation with *R. irregularis*. Colonization rates are estimated by quantifying the presence of AMS-induced pigment in *M. paleacea* apexes. Predicted protein structures are indicated on the left for the first exon. Red asterisks indicate the presence of a premature stop codon in the mutant line. *** and ** indicate statistical difference (p<0,001 and p<0.05 respectively) calculated with a pairwise comparison of proportions (Chi2) to the control line and a BH p-value adjustment. One out of three independent assays is shown, numbers of observed thalli in the assay are indicated (n).

C. Transversal sections of *M. paleacea cyclops* and control lines eight weeks post inoculation with *R. irregularis*. *R. irregularis* is visualized with WGA-Alexa Fluor 488. For each line, bright field, Alexa Fluor 488 and overlay are shown. Presence of the fungi was checked at cellular level by confocal microscopy (close-up pictures Alexa Fluor 488 and overlays). Scale bar=100µm (Nikon microscopy) and 10µm (confocal microscopy). *R. irregularis* is observed inside all thalli of all lines except *cyclops #3.9* and *cyclops #3.4*. On the right panel, the bars correspond to the expression levels of *MpaSymPT*, *MpaSTR* and *RiTEF* analyzed by qRT–PCR on thalli five weeks post inoculation with *R. irregularis*. Data were normalized to *M. paleacea* housekeeping genes *MpaEF1* and *MpaAPT2*. Error bars represent SE (n = 3 biological replicates). Asterisks indicate statistically significant differences (Student's t-test: ∗ p-value ≤ 0.1, ∗∗ p-value ≤ 0.05, and ∗∗∗ p-value ≤ 0.001) compared with the control lines.

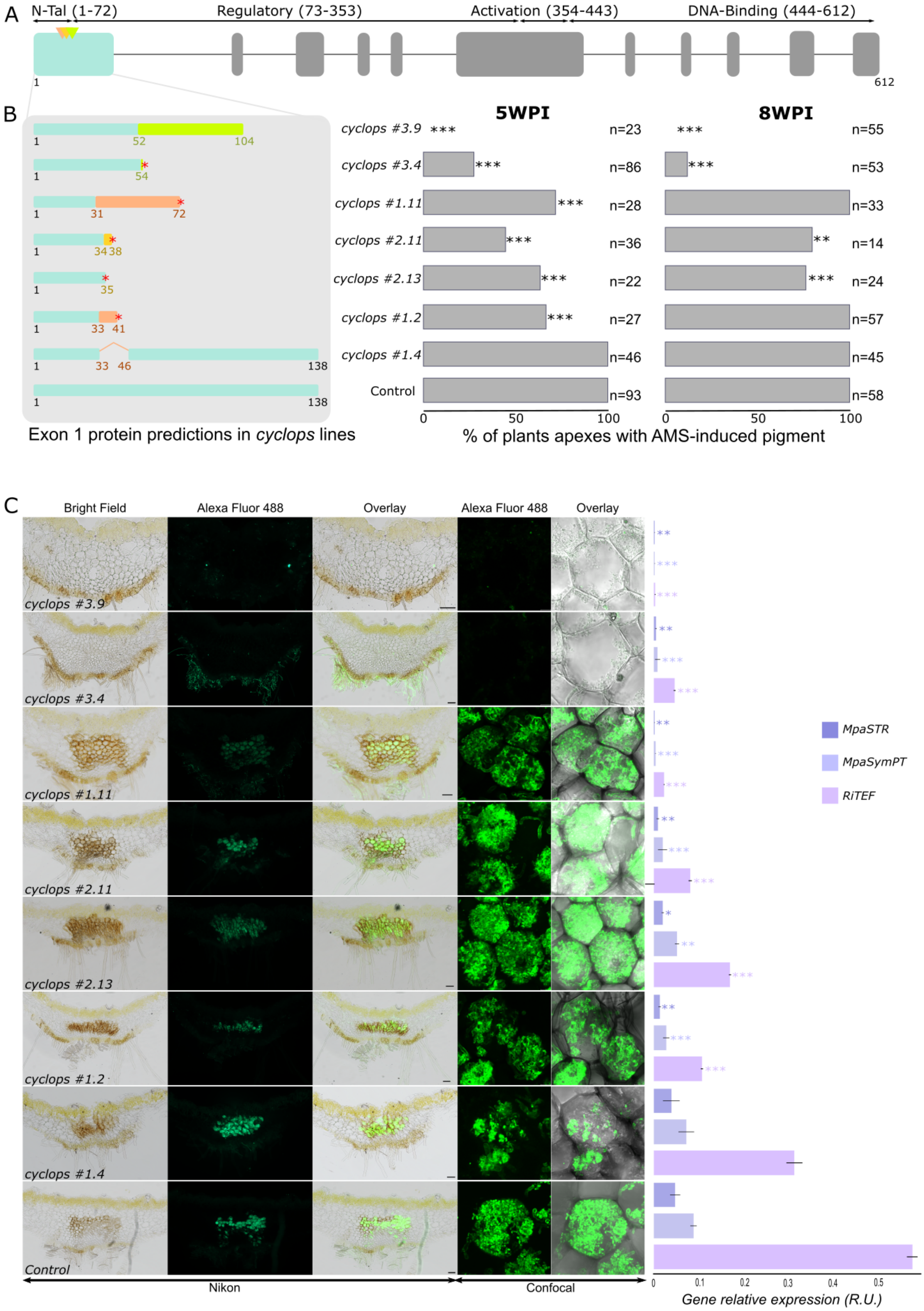

Supplemental Figure 8: Loss of a N-terminal domain in angiosperms CYCLOPS proteins

CYCLOPS phylogeny reconstructed using maximum likelihood. The untrimmed alignment of the proteins is shown next to the phylogeny. The N-terminal domain is absent (or highly divergent) in angiosperms and is highlighted in green. The regulatory, activation, and DNA-binding domains are highlighted in red, yellow, and blue, respectively. The two phosphorylation sites are shown with a dark blue rectangle; they were both mutated to D in CYCLOPS-DD overexpressing lines. Ultrafast bootstrap support (%; out of 1,000 replicates) is shown at the end of the branches. The scale represents the estimated number of substitutions per site. Sequences that differ from the original annotation are shown with an asterisk (*), and experimentally validated proteins are marked with a yellow star.

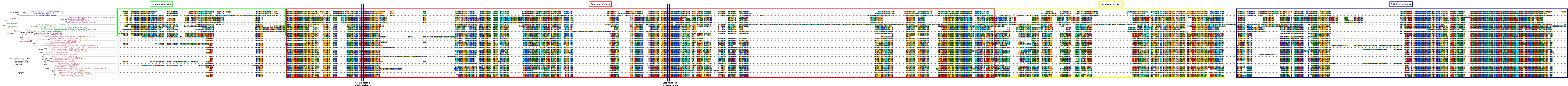

Supplemental Figure 9: *M. paleacea ssp diptera cyclops* lines are impaired for arbuscular mycorrhization with *R. irregularis*

A. Protein sequence alignment of MpaCYCLOPS first exon in wild type and *M. paleacea ssp paleacea (cyclops #1,2,3)* and *ssp diptera (cyclops #4)* mutant lines. The N-terminal domain absent from angiosperms is indicated in green, regulatory domain in black. Alignments were performed using Clustal Omega and visualized in AliView.

B. Colonization of *M. paleacea ssp diptera* lines five weeks post inoculation with *R. irregularis* is indicated. Colonization rates are estimated by quantifying the presence of AMS-induced pigment in *M. paleacea* apexes. Wild type parentale female was used as Control.*** indicates statistical difference (p<0,001) calculated with a pairwise comparison of proportions to the control line and a BH pvalue adjustment. n= number of observed thalli. One assay out of three independent assays is shown.

C. Sections of *cyclops* and control lines five weeks post inoculation with *R. irregularis*. *R. irregularis* is visualized with blue ink. Black arrows, white arrows and arrowheads indicate arbuscules, hyphae and vesicules respectively. Scale bar=100µm.

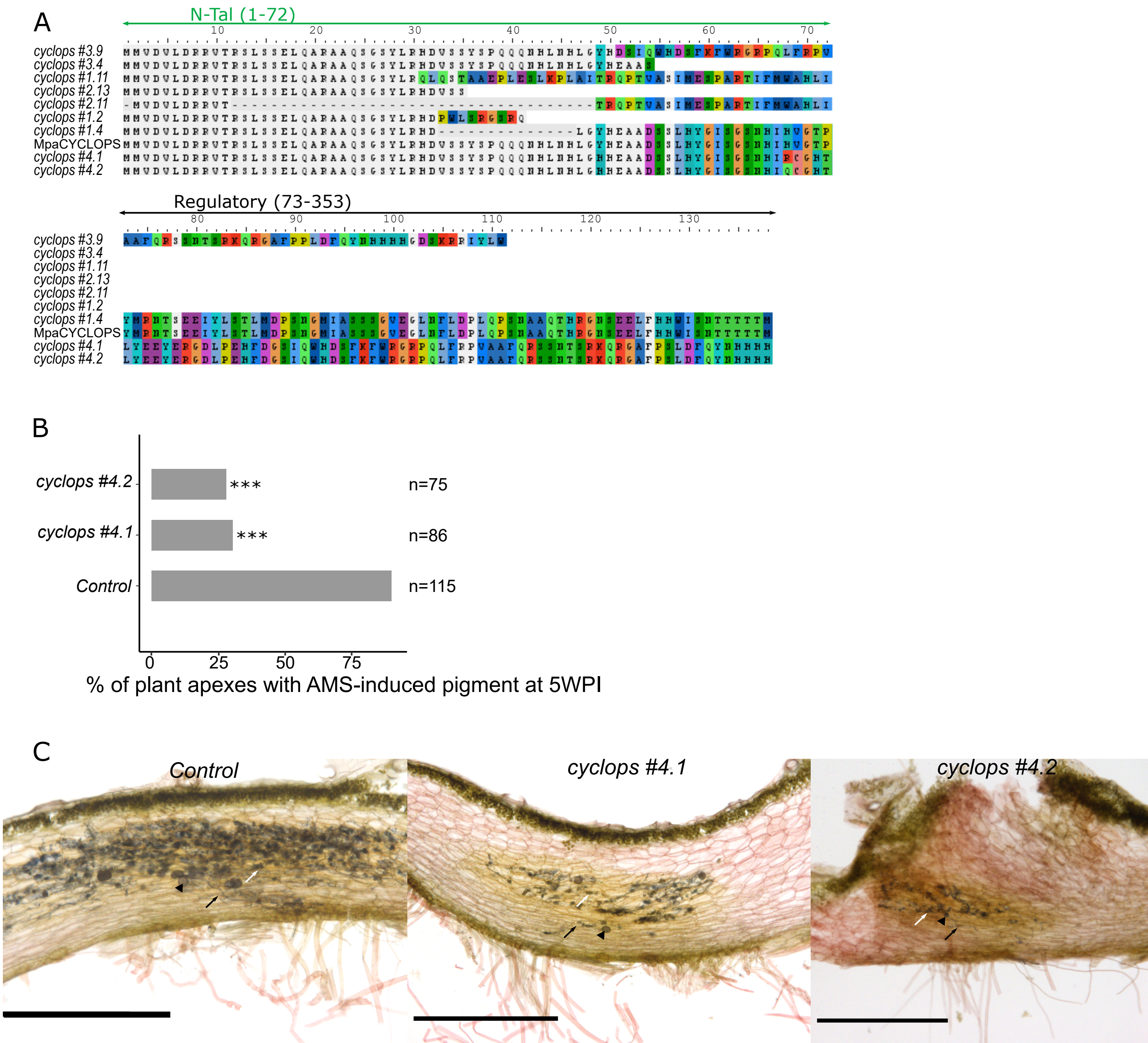

<u>Supplemental Table S1.</u> Number of plants showing root mycorrhizal arbuscules in *petunia* lines *ccamk* , *cyclops* and wild type. Total number of plants observed from three independent assays are shown

Line	AMS+
Wild Type	12/13
ccamk #1	0/9
cyclops #1	0/13

AMS + : Number of plants showing arbuscular mycorrhizal structures out of total plants observed.

Table S2. **List of primers for CRISPR lines.**

Gene	Primer	Sequence	Experiment
SYMRK	PMD1101	5'-cataGGTCGCCATTTGGTTACAAG	Guide RNA
SYMRK	PMD1102	5'-aaacCTTGTAACCAAATGGCGACC	Guide RNA
SYMRK	PMD1103	5'-cataGATCTCGTTCTAGCTGTGAAC	Guide RNA
SYMRK	PMD1104	5'-aaacGTTCACAGCTAGAACGAGATC	Guide RNA
SYMRK	PMD1105	5'-cataGATGATAGATACGTTCAGTC	Guide RNA
SYMRK	PMD1106	5'-aaacGACTGAACGTATCTATCATC	Guide RNA
SYMRK	PMD1107	5'-aaacGGCTTGTTTGGATGCCCGATC	Guide RNA
SYMRK	PMD1108	5'-cataGATCGGGCATCCAAACAAGCC	Guide RNA
SYMRK	PMD1209	5'-ACTTGTGGAGCAAATGTTGGGTAG	Genotyping
SYMRK	PMD1210	5'-ACTCATGCACGTGACAGTCGTC	Genotyping
CCAMK	PMD1125	5'-aaacCCATCGGACTCTTCCTCTTC	Guide RNA
CCAMK	PMD1126	5'-cataGAAGAGGAAGAGTCCGATGG	Guide RNA
CCAMK	PMD1127	5'-aaacCCCGGCCAGCTCGGGAGGAGC	Guide RNA
CCAMK	PMD1128	5'-cataGCTCCTCCCGAGCTGGCCGGG	Guide RNA
CCAMK	PMD1129	5'-cataGATGTCTACGAAGACCCGAC	Guide RNA
CCAMK	PMD1130	5'-aaacGTCGGGTCTTCGTAGACATC	Guide RNA
CCAMK	PMD1131	5'-cataGAGATGAACTACGTTCGCATG	Guide RNA
CCAMK	PMD1132	5'-aaacCATGCGAACGTAGTTCATCTC	Guide RNA
CCAMK	PMD1133	5'-aaacCCAGTGACCACATATCGCTGC	Guide RNA
CCAMK	PMD1134	5'-cataGCAGCGATATGTGGTCACTGG	Guide RNA
CCAMK	PMD1135	5'-aaacTGCTACCAGCCAGCGATATGC	Guide RNA
CCAMK	PMD1136	5'-cataGCATATCGCTGGCTGGTAGCA	Guide RNA
CCAMK	PMD1216	5'-TCAGCATGTATCCTACAGCACAGC	Genotyping
CCAMK	PMD1217	5'-TTTGGGGCTTAGGACTCTTTCAGG	Genotyping
CCAMK	PMD1476	5'-CAGAGAAGATGGAGCGCTTGTGG	Genotyping
CCAMK	PMD1477	5'-TGAGCTTGGTGTAGACTGGCGA	Genotyping
CYCLOPS	PMD534	5'-aaacCTTTCTGCTCCTCGGAGACAC	Guide RNA
CYCLOPS	PMD535	5'-cataGTGTCTCCGAGGAGCAGAAAG	Guide RNA
CYCLOPS	PMD536	5'-aaacCTGGAGGCGGTTGTCTCCGAC	Guide RNA
CYCLOPS	PMD537	5'-cataGTCGGAGACAACCGCCTCCAG	Guide RNA
CYCLOPS	PMD538	5'-aaacTGGCTTCGGCGGGAGCGACTC	Guide RNA
CYCLOPS	PMD539	5'-cataGAGTCGCTCCCGCCGAAGCCA	Guide RNA
CYCLOPS	PMD540	5'-cataGTGCCACCGCTTTCTGCTCCT	Guide RNA
CYCLOPS	PMD541	5'-aaacAGGAGCAGAAAGCGGTGGCAC	Guide RNA
CYCLOPS	PMD546	5'-aaacCAGCGCAAGGAGGCATTTTGC	Guide RNA
CYCLOPS	PMD547	5'-cataGCAAAATGCCTCCTTGCGCTG	Guide RNA
CYCLOPS	PMD548	5'-aaacCTGCCAAGGACAGAATCAGC	Guide RNA
CYCLOPS	PMD549	5'-cataGCTGATTCTGTCCTTGGCAG	Guide RNA
CYCLOPS	PMD1046	5'-ATGATGGTAGACGTTCTGGATA	Genotyping
CYCLOPS	PMD694	5'-TGGTGGTGGTGGTTGTATTG	Genotyping

Dataset S1 (separate file). RNAseq raw count data (A) and list of differentially regulated genes (B) in response to CYCLOPS-DD and CCaMK-Kin overexpression in M. paleacea. C. Statistical test of the overlap between CYCLOPS-DD and CCaMK-Kin-induced transcriptomic changes.

Dataset S2 (separate file). List of constructs used in this study.
